# Optimally Configured Optical Fiber Near‐Field Enhanced Plasmonic Resonance Immunoprobe for the Detection of Alpha‐Fetoprotein

**DOI:** 10.1002/advs.202207437

**Published:** 2023-03-30

**Authors:** Jianying Jing, Kun Liu, Junfeng Jiang, Tianhua Xu, Lu Xiao, Xiaohan Zhan, Tiegen Liu

**Affiliations:** ^1^ School of Precision Instruments and Opto‐Electronics Engineering Tianjin University Tianjin 300072 China; ^2^ Key Laboratory of Opto‐Electronics Information Technology Ministry of Education Tianjin University Tianjin 300072 China; ^3^ Tianjin Optical Fiber Sensing Engineering Center Institute of Optical Fiber Sensing Tianjin University Tianjin 300072 China; ^4^ School of Engineering University of Warwick Coventry CV4 7AL UK

**Keywords:** alpha‐fetoprotein, low detection limit, near‐field enhancement, optical fiber plasmonic immunoprobes, spectrum optimization, tumor early screening

## Abstract

The detection of trace biomarkers is an important supplementary approach for early screening and diagnoses of tumors. An optical fiber near‐field enhanced plasmonic resonance immunoprobe is developed for the detection of the hepatocellular carcinoma biomarker, i.e., the alpha‐fetoprotein. Generic principles based on dispersion models and finite element analysis (FEA) models are developed to realize the optimized configuration of spectral characteristics of the immunoprobe. Dispersion models provide theoretical guidance for the design of the multilayer sensing structure from the perspective of the ray optics theory. FEA models provide theoretical guidance for the selection of coating materials from the perspective of the self‐defined dielectric constant ratio, i.e., the ratio of the real part to the imaginary part. The optimized configuration of the antibody coupling further improves the biosensing performance of the immunoprobe. The limit of detection (LOD) can reach down to 0.01 ng mL^−1^, which is one order of magnitude lower than those relevant reported works. Such a low LOD can more effectively avoid the accuracy degradation of detection results due to measurement errors. Human serum samples have also been detected, with the good precision achieved. This work shows promising prospects in applications of label‐free, low‐cost, rapid, and convenient early screening of tumors.

## Graphical abstract

The design and the development of an optimized immunoprobe for the detection of the hepatocellular carcinoma biomarker are presented. The detection limit of the immunoprobe can reach down to 0.01 ng mL^−1^. Human serum samples are also been detected, with the good precision achieved. This work shows promising prospects in applications of label‐free, low‐cost, plug‐and‐play early screening of tumors.

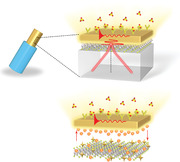

## 1 Introduction

The approaches for the tumor screening and diagnosis are mainly categorized into the imageological examination and the detection of serological tumor markers. The imageological examination methods such as the ultrasonic diagnosis^[^ ^1^ ^]^ can provide visualized results, but these methods rely on the phenotypic properties of tumors. This makes it difficult to distinguish sub‐centimeter‐sized tumors, and thereby their application in the early screening for tumors is limited. Therefore, the detection of tumor markers has gradually become an important supplementary method to imageological diagnoses. Clinically high‐specific tumor markers include the alpha‐fetoprotein (AFP), the prostate specific antigen (PSA), the carcino‐embryonic antigen, the cancer antigen 15–3, and the cancer antigen 12–5, etc. Among them, AFP (molecular weight 70 kDa) is a typical serological biomarker glycoprotein with high specificity and sensitivity for the diagnosis and the treatment monitoring of the primary hepatocellular carcinoma (HCC). HCC is the sixth most common malignant tumor, and its mortality is ranked in the fourth place over the world.^[^ ^2^ ^]^ The earlier the tumor is detected, the greater the chance of a cure and the better the prognosis for patients. Currently popular approaches for the detection of the AFP include the radioimmunoassay,^[^ ^3^ ^]^ the enzyme‐linked immunosorbent assay,^[^ ^4^ ^]^ the electro‐chemiluminescence immunoassay,^[^ ^5^ ^]^ the nanoparticle magnetic chemiluminescent immunoassay,^[^ ^6^ ^]^ etc. These methods can achieve the detection of the AFP at the level of ng mL^−1^ to fg mL^−1^, and relevant kits have been applied in the clinical medicine. However, these methods require the large and expensive equipment and will also introduce the radioactivity as well as the labeling of the fluorescence, the enzyme and the nanoparticle. These could lead to high costs and degradations in activities of antibodies.

Optical fiber plasmonic resonance technologies have advantages of high sensitivity, compact size, low cost, good flexibility, and simple platform, and can achieve the remote, on‐line, real‐time, label‐free, and multiparameter detection.^[^ ^7^, ^8^, ^9^ ^]^ The performance improvement approaches for the optical fiber plasmonic resonance based on multilayer sensing structures^[^ ^10^ ^]^ and various coating materials (including metal layer^[^ ^11^ ^]^ and dielectric layer^[^ ^12^ ^]^) have been widely reported. Aiming at the complexity of designing sensing structures and the diversity of selecting coating materials, a generic principle is required to guide the development of optical fiber plasmonic resonance sensors to achieve the optimization of spectral characteristics. Optical fiber plasmonic resonance sensors can realize the detection of multiple types of protein biomarkers by immobilizing corresponding types of antibodies on surfaces of the sensors via antibody coupling agents.^[^ ^13^ ^]^ The biocompatibility of the antibody coupling agent is the medium for converting the high sensitivity of the sensor into the spectral signal representing the microscopic coupling between protein molecules. Therefore, the optimization of types of antibody coupling agents and coupling conditions needs to be explored. The optimized configuration of spectral characteristics and the antibody coupling is aimed at improving the biosensing performance of the optical fiber plasmonic resonance sensor to realize the detection of trace biomarkers such as AFP.

Based on above discussions, generic principles based on dispersion models and finite element analysis (FEA) models are proposed to provide theoretical guidance for the design of multilayer sensing structures and the selection of coating materials, respectively. According to the guidance, a near‐field electronic transfer and coupling enhanced optical fiber plasmonic immunoprobe with a sensing structure of fiber core/WSe2 doped with AuNSs (WSe_2_@Au) nanostars (AuNSs) layer/Au layer is developed for the detection of the AFP. The high carrier mobility of the WSe_2_ layer and the electronic coupling arising from the tip hot spot in the nanogap between the Au nanostar and the Au layer synergistically realize the near‐field enhancement. This thereby improves the refractive index (RI) sensitivity of the immunoprobe.^[^ ^14^ ^]^ The designed sensing structure of fiber core/intermediate dielectric layer/metal layer confines the resonance dip within a short wavelength band and narrows the full width at half‐maximum (FWHM). This allows a larger amount of dopamine with better biocompatibility to be modified and this thereby further improves the biosensing performance of the immunoprobe. This work is focused on (1) generic principles for the optimized configuration of spectral characteristics of the optical fiber plasmonic resonance and (2) the biosensing performance of the immunoprobe. The developed immunoprobe exhibits a better performance and an easier operation in the detection of the AFP compared to reported works based on the fiber‐optic plasmon resonance.

## 2 Results and Discussion

### 2.1 Principles for the Optimized Configuration of Spectral Characteristics

Many types of multilayer sensing structures and coating materials are available for the development of optical fiber plasmonic resonance sensors.^[^ ^8^ ^]^ This section is focused on how to design the sensing structure and employ coating materials to optimize spectral characteristics of the optical fiber plasmonic resonance.

#### 2.1.1 The Design of Multilayer Sensing Structure of the Immunoprobe

Dispersion models have been built to demonstrate determinants of three intrinsic performance indicators of the optical fiber plasmonic resonance, including the resonance wavelength, the FWHM and the sensitivity. Detailed discussions can be found in Section S1 in the Supporting Information. According to dispersion models, a complex multilayer sensing structure is equivalent to a three‐layer sensing structure. The optimized configuration of the resonance wavelength and the FWHM can be achieved by configuring the locations and the number of intersections between Equations S1 and S2 (Supporting Information), as shown in Figures S1 and S3 (Supporting Information), respectively. The optimized configuration of the sensitivity can be achieved by the reasonable selection of coating materials. This provides a generic tool for the theoretical analyses of the wavelength‐modulated optical fiber plasmonic resonance.

**Figure 1:**
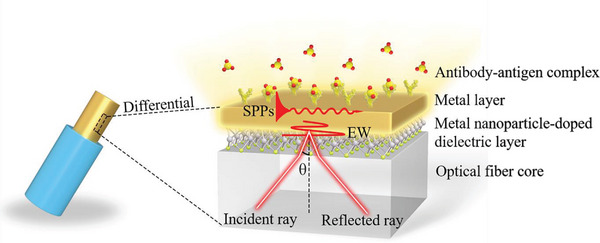
Schematic of the sensing structure of the optical fiber NFE‐PR immunoprobe.

Based on above discussions, the sensing mechanism of the optical fiber immunoprobe is based on the near‐field enhanced plasmonic resonance (NFE‐PR). This is hereafter referred to as the optical fiber NFE‐PR immunoprobe. In order to further enhance the sensitivity of the immunoprobe without significantly widening the FWHM, a small proportion of metal nanoparticles can be doped into the intermediate dielectric layer. The near‐field electronic coupling^[^ ^15^ ^]^ in hot spots between metal nanoparticles and the metal layer will lead to a significant enhancement of the surface electric field. Therefore, the designed sensing structure of the immunoprobe is optical fiber core/metal nanoparticle‐doped dielectric layer/metal layer/sample layer, as shown in **Figure** **1**.

#### 2.1.2 The Selection of Coating Materials of the Immunoprobe

FEA models have been implemented to investigate the influence of electromagnetic properties of coating materials on spectral characteristics of the optical fiber plasmonic resonance, including the resonance wavelength, the FWHM, the sensitivity, and the figure of merit (FOM). Detailed discussions can be found in Section S2 in the Supporting Information. According to FEA models, the FOM of the optical fiber plasmonic resonance can be preliminarily estimated by the dielectric constant ratio (DCR) of coating materials (especially metallic materials and 2D nanomaterials), rather than the use of the complex spectral calculation. The optimized configuration of the DCR can be achieved by the reasonable selection of coating materials and the modification of coating materials at the atomic level.^[^ ^16^ ^]^

The FOM of the Ag layer‐based fiber plasmonic resonance is the highest due to the DCR of the Ag layer being farthest from 1, as shown in Figure S7d in the Supporting Information. The sensitivity and the FWHM of the Ag layer‐based fiber plasmonic resonance are also higher and smaller than those of fiber plasmonic resonances stimulated by other metal layers, respectively. The reason is that the real part and the imaginary part of the dielectric constant of the Ag layer are smaller than those of other metal layers for the wavelength range of 500–800 nm. This leads to a larger penetration depth and propagation distance of surface plasmon polaritons (SPPs), respectively, according to Ref. [17]. However, the Ag layer is more susceptible to the oxidability, this thereby will lead to the performance degradation of the plasmonic resonance. The sensitivity and the FOM of the Au layer‐based fiber plasmonic resonance are ranked in second place, and the chemical stability of the Au layer is excellent. Therefore, the Au layer is more suitable as the metal layer in the development of the optical fiber NFE‐PR immunoprobe.

**Figure 2:**
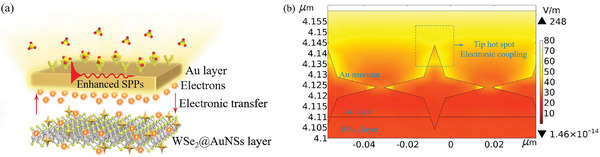
a) Schematic of coating materials of the optical fiber NFE‐PR immunoprobe. Red arrows represent the electronic transfer between the Au layer and the WSe_2_ layer. The aggregation of electrons in the region near the Au layer represents the near‐field enhancement. b) The mode‐field distribution of the tip hot spot obtained by the FEA analysis. The electric field intensity on the upper surface of the Au layer can reach up to 77.38 V m^−1^.^[^ ^14^ ^]^

In the wide RI range of 1.33–1.38, the FOM of the WS_2_‐modified fiber plasmonic resonance is the highest due to the largest DCR of the WS_2_ layer, as shown in Figure S12d in the Supporting Information. However, the sensitivity of the WSe_2_‐modified fiber plasmonic resonance is the highest in the RI range of 1.33–1.34 corresponding to that of the biobuffer‐prepared protein sample with a concentration on the order of nanogram per milliliter, as shown by enlarged columns in Figure S12b in the Supporting Information. The FOM of the WSe_2_‐modified fiber plasmonic resonance is similar to that of the WS_2_‐modified fiber plasmonic resonance. In addition, the carrier concentration and the carrier mobility of the WSe_2_ layer are 1–2 orders of magnitude higher than those of other transition metal dichalcogenide (TMDC) layers.^[^ ^14^ ^]^ Compared to other types of metal nanoparticles, AuNSs possess good stability and can produce more significant electric‐field enhancement due to the tip hot spot effect.^[^ ^14^, ^18^ ^]^ Therefore, the composite layer of WSe_2_ and AuNSs (WSe_2_@AuNSs layer) is more suitable as the dielectric layer in the development of the immunoprobe. The selection of coating materials and the tip hot spot effect are shown in **Figure** **2**a,b, respectively.

### 2.2 Immunoassay with the Developed Immunoprobe

Based on above principles for guiding the design of multi‐layer sensing structures and the selection of coating materials, optical fiber NFE‐PR immunoprobes are practically fabricated for the detection of the AFP.

#### 2.2.1 Detection of PBS‐Prepared AFP Samples

**Figure 3:**
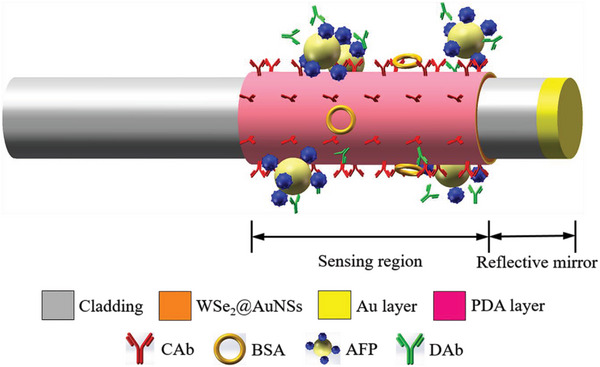
Schematic of the double‐antibody sandwich immunoassay for the AFP using the optical fiber NFE‐PR immunoprobe.

**Figure 4:**
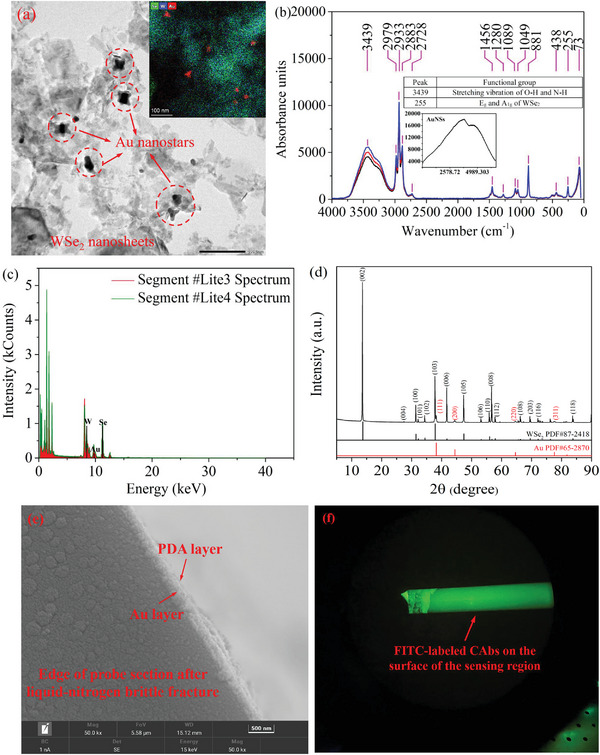
a) Transmission electron microscope (TEM) image of the WSe_2_@AuNSs composite layer. Inset: high angle annular dark field scanning transmission electron microscopy (HAADF‐STEM) image of the composite layer. b) Three measurements of Raman spectra of the WSe_2_@AuNSs composite layer. There is no characteristic peak of AuNSs, and the Raman signal of AuNSs is shown in the inset. c) Two energy dispersive spectrometer (EDS) spectra of the WSe_2_@AuNSs composite layer. d) The X‐Ray diffraction (XRD) measurement of the WSe_2_@AuNSs composite layer, including the phase identification and the crystal face marking. e) The scanning electron microscope image of the PDA layer on the sensing region. f) The fluorescence microscope image of the sensing region coated by fluorescein isothiocyanate (FITC)‐labeled CAbs.

The detection of the AFP via the immunoprobe is based on the double‐antibody sandwich immunoassay, and the proposed scheme is shown in **Figure** **3**. The characterizations of coating materials in the immunoprobe can be seen in **Figure** **4**. Double‐antibody sandwich immunoassay is a typical signal enhancement method in the immunodetection employing plasmonic resonance sensors. The introduction of a secondary antibody can increase the molecular density on the surface of the sensing region.^[^ ^19^ ^]^ This thereby further improves the limit of detection (LOD). The LOD of the immunoprobe aims to meet the requirement of the low‐cost early screening of tumors. It is promising to achieve an ultralow LOD by using a low dimensional nanomaterial‐labeled secondary antibody as a signal enhancer.^[^ ^20^, ^21^ ^]^ This will also increase the difficulty and the cost in the development of the sensor. Therefore, the secondary antibody (i.e., the detection antibody (DAb)) in our work is unlabeled, and it will not significantly increase the complexity of the detection due to the plug‐and‐play probe structure.

**Figure 5:**
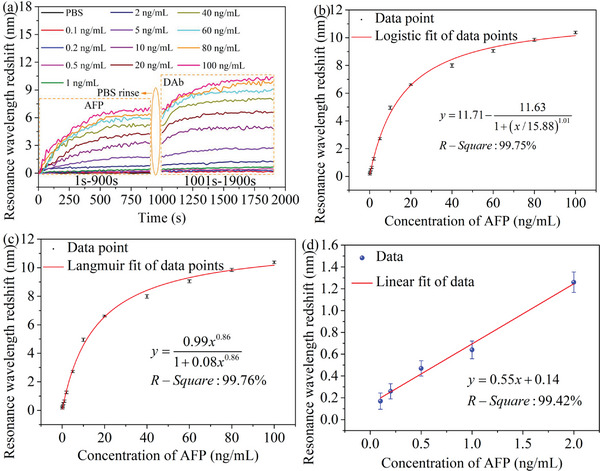
a) Redshifts of resonance wavelengths with the increment of the time when AFP samples with different concentrations are detected. b) Logistic and c) Langmuir dynamic calibration curves of the optical fiber NFE‐PR immunoprobe. d) The linear fitting of the resonance wavelength redshifts and the concentrations in the range of 0.1–2 ng mL^−1^. The data point is the mean value of the resonance wavelength redshift over the last 50 s (1851–1900 s). The error bar is the standard deviation from the mean of redshifts obtained by using three immunoprobes from one batch and with similar performance to detect the same sample.

The optimized configuration of antibody coupling agents and coupling conditions has been first investigated in Section S3 in the Supporting Information. The optical fiber NFE‐PR immunoprobe with dopamine‐immobilized capture antibodies (CAbs) is inserted into an AFP sample of 5 ng mL^−1^, and the initial resonance wavelength is immediately recorded (blue curve in Figure S13b in the Supporting Information). As the combining between the CAbs and the AFP (in Figure S18d in the Supporting Information), the resonance wavelength exhibits a redshift, as shown in **Figure** **5**a (1–900 s). After 15 min, the immunoprobe is gently rinsed by the phosphate buffer saline (PBS) and inserted into the DAb solution with a concentration of 500 ng mL^−1^ for 15 min. The resonance wavelength again shows a redshift relative to the initial resonance wavelength in the DAb solution due to the interaction between the DAbs and the AFP (in Figure S18e in the Supporting Information), as seen in Figure 5a (1001–1900 s). The final resonance wavelength is also recorded (magenta curve in Figure S13b in the Supporting Information). The volume of the AFP sample is 4 mL, which is within the volume range (commonly 2–15 mL) of the serum used in the clinical detection. The AFP samples with different concentrations are detected by immunoprobes which are fabricated in the same batch and have similar sensing performance. This avoids the degradation of the sensing performance of the immunoprobe due to the dissociation and wash of the antigen–antibody complex when the samples with different concentrations are measured by the same sensing element. The discussion on the intrabatch difference of immunoprobes can be found in Section S4.1 in the Supporting Information.

The calibration for the optical fiber NFE‐PR immunoprobe can be achieved via the detection of a finite number of PBS samples. Both the Logistic model^[^ ^22^ ^]^ and the Langmuir^[^ ^23^ ^]^ model are common for bioaffinity reactions in clinical applications. Therefore, the calibration curves of the immunoprobe based on above two models are shown in Figure 5b,c, respectively. A resonance wavelength redshift can be obtained by using an immunoprobe to detect a sample with an unknown concentration and can also be regarded as the *y*‐coordinate in the calibration curve. The concentration of the sample is shown in the *x*‐coordinate corresponding to the *y*‐coordinate in the calibration curve. The sensitivity of the immunoprobe can be obtained by linearly fitting the first five data points with good linearity and close to the zero concentration,^[^ ^24^ ^]^ as shown in Figure 5d. The slope of the fitted line represents the sensitivity of the immunoprobe, which is 0.55 nm (ng mL^−1^)^−1^. The LOD of the immunoprobe is calculated by Equation 1 ^[^ ^25^ ^]^

(1) $$\mathrm{LOD}=f^{-1}\left({\bar{y}}_{\mathrm{blank}}+3\sigma_{\max}\right)$$

where *f*(*x*) is the calibration function. ${\bar{y}}_{\mathrm{blank}}$ is the mean value of the resonance wavelength redshift obtained by detecting the blank sample (i.e., pure PBS, black curve in Figure 5a). *σ*_max_ is the maximum standard deviation of the resonance wavelength redshift over the last 50 s in Figure 5a. The LODs of the immunoprobe calculated by the Logistic and the Langmuir calibration principles are 0.0111 ng mL^−1^ (0.1586 × 10^−12^ m) and 0.0549 ng mL^−1^ (0.7843 × 10^−12^ m), respectively.

**Figure 6:**
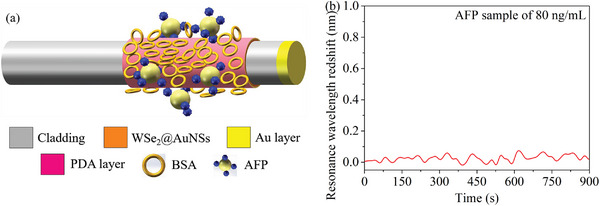
a) The sensing structure of the fully BSA‐passivated immunoprobe. b) The resonance wavelength redshift obtained by detecting the AFP sample of 80 ng mL^−1^ via the fully BSA‐passivated immunoprobe. The final redshift is 0.0358 nm calculated from the mean value of redshifts over the last 50 s.

In order to investigate the influence of the nonspecific deposition of the antigen on above detection results, a fully bovine serum albumin (BSA)‐passivated immunoprobe has been fabricated to detect the AFP sample. After the self‐polymerization of the dopamine onto the surface of the immunoprobe, the surface of the immunoprobe is directly blocked by the BSA and unmodified with the antibody. In order to observe a more obvious redshift signal, the concentration of the AFP sample is 80 ng mL^−1^. The demodulated redshift of the resonance wavelength is 0.0358 nm, as shown in **Figure** **6**. The redshift is within the range of the error caused by demodulation algorithms and the intrabatch difference of immunoprobes. This indicates that the nonspecific deposition of the antigen produces a marginal error in the resonance wavelength redshift arising from the specific interaction. A supplementary experiment on the introduction of the PBS immersion after the detection of the AFP has also been implemented to support the above conclusion, as seen in Section S4.2 in the Supporting Information.

Previously reported works on the detection of the AFP using fiber plasmonic resonance technologies associated with our developed method have been listed in **Table** **1**. The LOD of the developed immunoprobe is approximately one order of magnitude lower than those of reported sensors. The upper limit of the clinical normal reference value of the AFP is 7–10 ng mL^−1^. The LOD of the immunoprobe is two orders of magnitude lower than the upper limit. Such a low LOD can avoid the risk of reducing the detection accuracy due to the temperature fluctuation, the intrabatch difference of the immunoprobe, etc. Additionally, the low‐cost and mass‐fabricated probe structure realizes the one‐off plug and play. The comparison demonstrates that the developed immunoprobe combines merits of the low LOD, the label‐free, and the easy operation.

**Table 1:** Comparison between the study of the AFP detection in this paper and reported works

Principle	Auxiliary means	Sensing structure	Detection range [ng mL^−1^]	Sensitivity [nm (ng mL^−1^)^−1^]	Fitting method for LOD	LOD [ng mL^−1^]	Ref.
Localized plasmonic resonance	Fluorescence	Transmission type	0.1–100	–	Linear fitting in a logistic scale	0.10	[26]
Localized plasmonic resonance	Label free	Transmission type	0.2–1000	–	Nonlinear fitting	0.20	[27]
Multi‐layered plasmonic resonance	Dual‐lane	Transmission type	25–400	0.06	Linear fitting	25	[28]
Localized plasmonic resonance	Label free	U‐bend type	5–200	–	Linear fitting	0.85	[29]
Near‐field enhanced plasmonic resonance	Label free	Probe type	0.1‐100	0.55	Logistic fitting	0.01	This work
					Langmuir fitting	0.05	

#### 2.2.2 Repeatability and Specificity of the Immunoprobe

**Figure 7:**
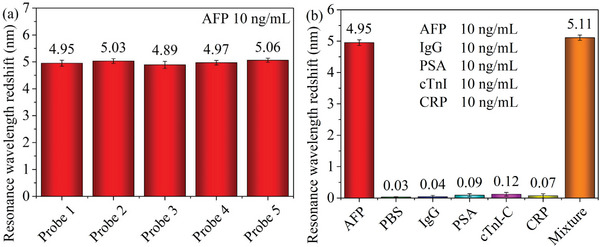
a) The repeatability and b) the specificity of the optical fiber NFE‐PR immunoprobe.

The repeatability of the plasmonic resonance biosensor is measured via the consistency of the results obtained from detecting the same target sample. The good repeatability represents the low cost of the biosensor and the high reliability of detection results. Five immunoprobes, fabricated from the same batch, with similar performance are used to detect the same AFP sample of 10 ng mL^−1^, and experimental results are shown in **Figure** **7**a. The obtained resonance wavelength redshifts have a standard deviation of 0.06 nm. This indicates a marginal intra‐batch difference and the good repeatability of the immunoprobes.

The specificity of the plasmonic resonance biosensor is measured by detecting the target sample and control samples. This aims to investigate the interference degree of control samples to the detection of the target sample. The good specificity represents the high precision of detection results. The topic of our work is focused on the detection of biomarkers for the early screening of human diseases. Therefore, the human immunoglobulin G (IgG, a biomarker antibody of immune diseases^[^ ^30^ ^]^), the human PSA (a biomarker of the prostate cancer^[^ ^31^ ^]^), the human cardiac troponin I‐C (cTnI‐C, a biomarker of the myocardial damage^[^ ^24^ ^]^), and the human c‐reactive protein (CRP, a biomarker of the cardiovascular inflammation^[^ ^32^ ^]^) are selected as controls. The PBS sample is used as the blank control, and the control sample of the mixture including the AFP, the IgG, the PSA, the cTnI‐C, and the CRP is employed to emulate complex components in the human serum. Seven immunoprobes, fabricated in the same batch, with similar performance are used to detect the target sample and control samples, and experimental results are shown in Figure 7b. The resonance wavelength redshifts corresponding to the AFP sample and the mixture sample behave similarly to the result in Figure 5a (the purple curve corresponding to 10 ng mL^−1^), and the redshifts corresponding to the samples without AFP are close to zero. This indicates that the control biomarkers will not have a significant interference on the detection of the AFP and the immunoprobes have good specificity.

Based on the good specificity of the immunoprobe, the detection of other types of high‐specific biomarkers can be achieved by modifying corresponding types of antibodies on the immunoprobe. This thereby will provide auxiliary approaches for the early screening of other types of tumors.

#### 2.2.3 Detection of Human Serum Samples

**Figure 8:**
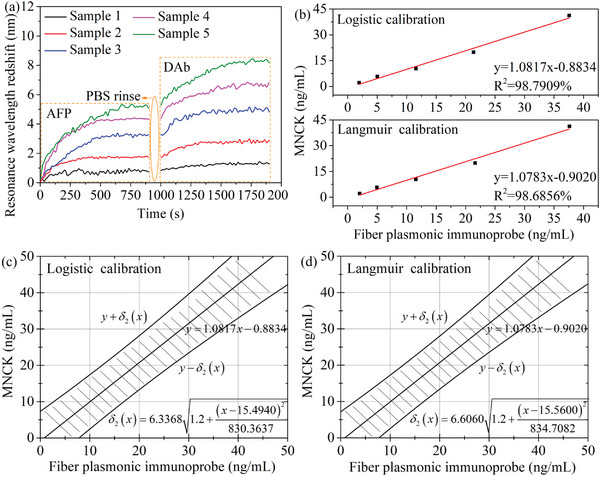
a) Redshifts of resonance wavelengths with the increment of the time when the serum samples with different concentrations of the AFP are detected. b) Correlation between the results of the optical fiber NFE‐PR immunoprobes and the MNCK for the detection of AFP in the human serum. The best‐fit line (solid) and the 95% prediction interval (dash) of c) the Logistic calibration and d) the Langmuir calibration, respectively.

Normal levels of the AFP in the human serum are ≈0–10 ng mL^−1^, and these levels can be elevated to more than 400 ng mL^−1^ in patients with HCC.^[^ ^33^, ^34^ ^]^ The calibration curves of the fiber immunoprobe are obtained in the concentration range of 0.1–100 ng mL^−1^ only. The detection of the serum samples with higher concentrations of the AFP can be achieved by diluting the samples with the normal saline. The high specificity of the fiber immunoprobe ensures the applicability of the calibration curves in the detection of serum samples due to the marginal influence of complex components in the human serum. The serum from healthy individuals with an original AFP concentration of 3.74 ng mL^−1^ has been collected from NovoBiotechnology Co., Ltd. Beijing, China. Five serum samples with concentrations ranging from ≈2 to 40 ng mL^−1^ are prepared by diluting the serum or spiking AFP into the serum. The developed optical fiber NFE‐PR immunoprobes in this work are used to detect the serum samples, and the redshifts of the resonance wavelengths are shown in **Figure** **8**a. Meanwhile, the magnetic nanoparticle chemiluminescence kit (MNCK) with a detection range of 1.80–1000 ng mL^−1^, purchased from Autobio Diagnostics Co., Ltd. Zhengzhou, China, has also been used to detect the serum samples. The linear regression analyses (in Section S5 in the Supporting Information) of the results obtained by the immunoprobes and the MNCK are shown in Figure 8b–d. The calculated correlation coefficients based on the Logistic calibration and the Langmuir calibration are 98.7909% and 98.6856%, respectively. The good correlation between the results obtained by above two approaches indicates the feasibility of the developed fiber immunoprobe in the real serum assay.

## 3 Conclusions

This work presents the design and the development of an optimized tool, i.e., an optical fiber near‐field enhanced plasmonic resonance immunoprobe, for the detection of the AFP which is a biomarker for the hepatocellular carcinoma. According to the dispersion models and the FEA models, the optimally configured spectral characteristics of the immunoprobe arise from (1) the near‐field electronic transfer and coupling in coating materials, (2) the better biosensing performance of the thicker polydopamine (PDA) layer, and (3) the accurate demodulation of spectral signals. The LODs of the immunoprobe can achieve down to 0.01 ng mL^−1^ (Logistic fitting) and 0.05 ng mL^−1^ (Langmuir fitting). Results demonstrate a better LOD level compared to reported similar studies and achieve good precision in prepared clinical samples. This work provides novel and insightful visions on the application of the plasmonic resonance optics method in the field of early screening and early diagnosis of tumors.

## 4 Experimental Section

### Materials and Reagents

The multimode fiber with a silica core of 600 µm, a plastic cladding of 630 µm, and a numerical aperture of 0.37 was purchased from Beijing Scitlion Technology Co., Ltd. Beijing, China. Dopamine hydrochloride (98% wt%), tris (hydroxymethyl) aminomethane (99% wt%), *N*‐(3‐dimethylaminopropyl)‐*N*′‐ethylcarbodiimide hydrochloride (EDC), and *N*‐hydroxysuccinimide (NHS) were purchased from Shanghai Aladdin Biochemical Technology Co., Ltd. Shanghai, China. Hydroxylated and carboxylated WSe_2_ nanosheet dispersion (1–10 layers, size: 50–1000 nm, 0.5 mg mL^−1^) and AuNSs dispersion (diameter: 40–45 nm, 0.1 mg mL^−1^) were purchased from Nanjing MKNANO Tech. Co., Ltd. Nanjing, China (www.mukenano.com). PBS (10 × 10^−3^ m, pH = 7.2–7.4), BSA, human AFP, polyclonal rabbit anti‐AFP (serving as the CAb), monoclonal mouse anti‐AFP (serving as the DAb), human IgG, human PSA, human cTnI‐C, and human CRP were purchased from Wuhan Huamei Biotech Co., Ltd. Wuhan, China.

### Fabrication of the Optical Fiber NFE‐PR Immunoprobe

(1) Coating of the dielectric layer and the metal layer—The end face of an optical fiber with a length of 10 cm was ground into a flat, and a part of the cladding of the optical fiber was removed, as seen in Figure S17a in the Supporting Information. The end face and the cladding‐removed part with a length of 1.2 cm were fabricated as the reflection region and the sensing region, respectively. The reflective mirror was fabricated by sputtering an Ag layer of 200 nm and an Au layer of 5 nm on the end face of the fiber core in turn, as seen in Figure S17b in the Supporting Information. A composite layer of hydroxylated WSe_2_@AuNSs (WSe_2_: AuNSs = 5:1, volume ratio) with a thickness of ≈10 nm was coated on the surface of the sensing region by the electrostatic self‐assembly,^[^ ^14^ ^]^ as seen in Figure S17c in the Supporting Information. Due to the saturation of the electrostatic adsorption, the thickness of the composite layer cannot be flexibly adjusted in a large range. An Au layer of 50 nm was then sputtered on the surface of the composite layer to excite the plasmonic resonance. At this point, the fabrication of an optical fiber probe was completed, as seen in Figure S17d in the Supporting Information.
(2) Characterization analyses of the WSe_2_@AuNSs layer—The transmission electron microscope (TEM) image and the Raman spectrum of the composite layer are shown in Figure 4a,b, respectively. The energy dispersive spectrometer (EDS) analysis and the X‐ray diffraction (XRD) measurement of the composite layer are shown in Figure 4c,d, respectively.
(3) Immobilization of the antibody—The sensing region of the fiber probe was immersed in a Tris‐prepared dopamine solution (2 mg mL^−1^, pH = 8.5) for 120 min, which was carried out in a thermostat steam bath vibrator. Under the alkaline condition, a PDA layer with active functional groups of the catechol quinone can be formed by the self‐polymerization of the dopamine. The PDA layer with strong hydrolysis resistance adhered tightly to the surface of the Au layer through complex physicochemical effects, e.g., hydrogen bonding, chelation, *π*–*π* interaction, and covalent bonding,^[^ ^35^ ^]^ as seen in Figure 4e and Figure S18a in the Supporting Information. The probe was then immersed into a PBS‐prepared CAb solution with a concentration of 2 µg mL^−1^ for 24 h at 4 °C. Quinones can be covalently coupled with amino‐terminated CAbs by the Schiff base or the Michael addition reaction,^[^ ^36^ ^]^ as seen in Figure S18b in the Supporting Information. Afterwards, the probe was rinsed by PBS to remove the free CAbs, and was immersed into a BSA solution with a concentration of 1 mg mL^−1^ to block nonspecific binding sites, as seen in Figure S18 in the Supporting Information. At this point, the fabrication of the optical fiber immunoprobe was completed. In order to demonstrate the effectiveness of the above antibody‐immobilization method, fluorescein isothiocyanate (FITC)‐labeled CAbs were immobilized on the surface of the sensing region, and the fluorescence microscope image of the sensing region is shown in Figure 4f. The green fluorescence indicated that the CAbs are evenly and densely distributed on the surface of the sensing region. The FITC‐labeled CAbs were used only for the demonstration, and the CAbs used in experiments are unlabeled.

### Experimental Setup

The experimental setup is shown in Figure S19 in the Supporting Information. The ray emitted from a deuterium‐halogen lamp (DH‐2000, Ocean Insight, Inc. Florida, USA, 215 nm‐2500 nm) entered the immunoprobe through a Y‐type fiber patch cord and excited the plasmonic resonance to sense the microscopic coupling between biomolecules. The ray was then reflected into the patch cord by the Ag mirror and collected by a spectrometer (Maya2000 Pro, Ocean Insight, Inc. Florida, USA, 165–1100 nm). The insertion of the immunoprobe into the sample and the withdrawal were controlled by a dip coater (SYDC‐100, Shanghai SAN‐YAN instrument Co., Ltd. Shanghai, China). The sample was placed in a waterbath (CW3‐05P, JeioTech Co., Ltd. Shanghai, China) with a constant temperature of 37 °C to promote the binding between the antigen and the antibody. Spectral signals were monitored via a computer in a real‐time manner. The noise of the resonance dip was suppressed and eliminated by the variational mode decomposition,^[^ ^37^ ^]^ as shown in Figure S20a in the Supporting Information. The resonance wavelength was demodulated by the weighted wavelength algorithm^[^ ^38^ ^]^ and denoised by the wavelet packet decomposition,^[^ ^37^ ^]^ as shown in Figure S20b in the Supporting Information.

## Conflict of Interest

The authors declare no conflict of interest.

## Supporting information

Supporting Information

## Data Availability

The data that support the findings of this study are available from the corresponding author upon reasonable request.
