# Supplementary material for: Optimally Configured Optical Fiber Near‐Field Enhanced Plasmonic Resonance Immunoprobe for the Detection of Alpha‐Fetoprotein
Source: Adv Sci (Weinh). 2023 Mar 30;10(15):2207437. doi: 10.1002/advs.202207437 (PMC10214261; doi:10.1002/advs.202207437)
Supplement: Supplementary file 1 — Supporting Information [file ADVS-10-2207437-s001.pdf]

ADVANCED SCIENCE
Open Access

Supporting Information

for *Adv. Sci.*, DOI 10.1002/advs.202207437

Optimally Configured Optical Fiber Near-Field Enhanced Plasmonic Resonance Immunoprobe for the Detection of Alpha-Fetoprotein

Jianying Jing, Kun Liu, Junfeng Jiang, Tianhua Xu, Lu Xiao, Xiaohan Zhan and Tiegen Liu*

Supporting Information

Optimally Configured Optical Fiber Near-Field Enhanced Plasmonic Resonance Immunoprobe for the Detection of Alpha-Fetoprotein

Jianying Jing, Kun Liu, Junfeng Jiang, Tianhua Xu, Lu Xiao, Xiaohan Zhan, Tiegen Liu*

This file includes:

Section S1: Dispersion Models of the Optical Fiber Plasmonic Resonance

Section S2: Finite Element Analysis Models of the Optical Fiber Plasmonic Resonance

Section S3: The Optimized Configuration of the Antibody Coupling

Section S4: The Discussion on the Not-specific Deposition of the Antigen

Section S5: Linear Regression Analysis

Supplementary Figures: Figures S17-S20

S1. Dispersion Models of the Optical Fiber Plasmonic Resonance

S1.1. Resonance Wavelength

The conventional plasmonic resonance (C-PR) is excited by a sensing structure of substrate/metal layer/sample layer. The substrate in our work is focused on the silica fiber core. The dispersion relationship between the phase constant k_{EW} of the evanescent wave (EW) produced by the incident ray and the wavelength can be expressed by Equation S1.[S1] The dispersion relationship between the phase constant k_{SPs} of surface plasmons (SPs) on the surface of the metal layer and the wavelength can be given by Equation S2.[S2]

$$\frac{\lambda_p}{\lambda} = \left(\sqrt{\varepsilon_o}\sin\theta\right)^{-1} \cdot \frac{k_{EW}}{k_p} \tag{S1}$$

$$\frac{\lambda_p}{\lambda} = \sqrt{\frac{\varepsilon_m + \varepsilon_s}{\varepsilon_m \cdot \varepsilon_s}} \cdot \frac{k_{SPs}}{k_p} \tag{S2}$$

where λ_p and λ are the plasma wavelength and the wavelength of the incident ray, respectively. ε_o, ε_m and ε_s are dielectric constants of the optical fiber core, the metal layer and the sample layer, respectively. θ is the incident angle of the incident ray. $k_p = \omega_p / c$ is the wavenumber. The dispersion relationship can be transformed into a dispersion model based on the simplified Drude model for the metal dispersion $\varepsilon_m = 1 - \omega_p^2 / \omega^2$,[S1] and Equations S1 and S2 are given as the red line and the magenta curve in **Figure S1**a, respectively. The generated magenta intersection indicates the excitation of the C-PR, as shown by Point 1 in **Figure S1**a. The corresponding wavelength λ in the y-coordinate of Point 1 represents the resonance wavelength λ_{res1} which can be found in **Figure S1**b.

Nearly guided wave plasmonic resonance (NGW-PR)[S1] can be excited by coating a dielectric layer with a high RI on the upper surface of the metal layer based on the C-PR. The dielectric layer and the sample layer can be considered as a hybrid sample layer with a higher equivalent RI (i.e., a higher ε_s) compared to that of the bare sample layer. This reduces the extreme value of Equation S2 (olive curve in **Figure S1**a), and the olive intersection indicates the excitation of the NGW-PR, as shown by Point 2 in **Figure S1**a. The corresponding resonance wavelength is located within a large wavelength band, as seen by λ_{res2} in **Figure S1**b. The additional modification of the antibody coupling agent (e.g., the dopamine) and the binding between antigens and antibodies can lead to a significant redshift and broadening of

the resonance dip.[S3] This may cause the resonance wavelength o exceed the wavelength band (typically 500 nm-1100 nm[S3]) where spectral signals can be demodulated accurately.

In order to confine the resonance wavelength within a wavelength band where it is convenient for the signal demodulation. The near-field enhanced plasmonic resonance (NFE-PR) can be excited by coating a plasmon-active dielectric layer with a high RI (typically > 2) between the fiber core and the metal layer. The dielectric layer and the fiber core can be regarded as a hybrid fiber core with a higher equivalent RI (i.e., a higher ε_o) compared to that (approximately 1.4580) of the bare core. This reduces the slope of Equation S1 (blue line in **Figure S1**a), and the blue intersection indicates the excitation of the NFE-PR, as shown by Point 3 in **Figure S1**a. The corresponding resonance wavelength is located within a short wavelength band, as seen by λ_{res3} in **Figure S1**b.

Values of resonance wavelengths can be calculated from y-coordinates of intersections in **Figure S1**a. Resonance wavelengths can also be calculated by fitting a transcendental Equation S3 obtained by the equality between Equations S1 and S2.[S4]

$$\lambda_{res} = \frac{2\pi c}{\omega_p}\sqrt{\frac{n_o^2 \sin^2\theta \cdot n_s^2 + n_o^2 \sin^2\theta - n_s^2}{n_o^2 \sin^2\theta - n_s^2}} \qquad \text{(S3)}$$

where c is the speed of the incident light (in vacuum), ω_p is the plasma frequency, n_o and n_s are refractive indices of the (hybrid) fiber core and the (hybrid) sample layer, respectively. The numerical calculation can realize the flexible and customized assignment of the wavelength band where the resonance wavelength is located aiming at a specific detection requirement.

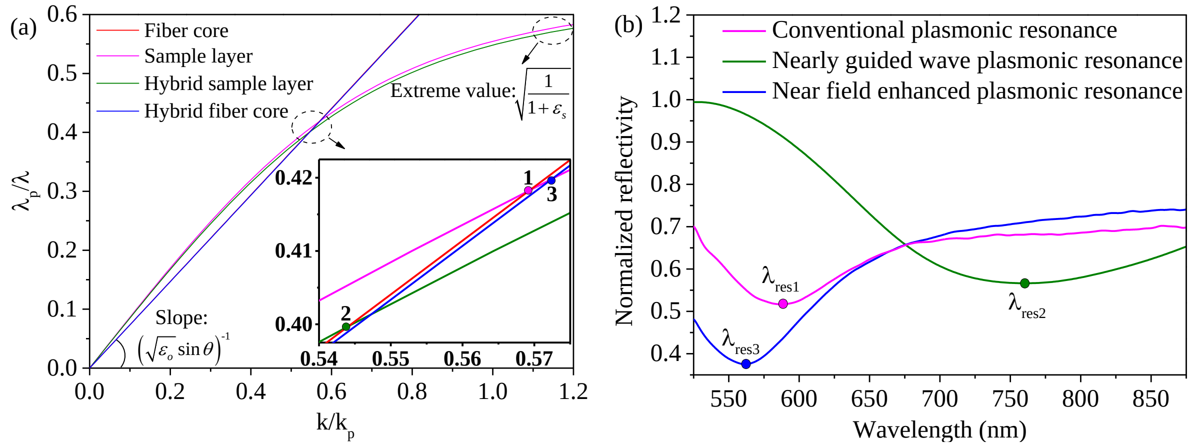

Figure S1. a) Dispersion models expressing resonance wavelengths of the C-PR (Point 1), the NGW-PR (Point 2) and the NFE-PR (Point 3). b) Resonance dips of the C-PR (λ_{res1}), the NGW-PR (λ_{res2}) and the NFE-PR (λ_{res3}).

S1.2. Full Width at Half-Maximum

The observed resonance dip of the optical fiber plasmonic resonance is the convolution of multiple resonance dips stimulated by separate EW modes.[S5] Therefore, the first determinant of the FWHM is the number of guided modes in the fiber that can excite the plasmonic resonance, i.e., the number of intersections between Equations S1 and S2. According to the transfer matrix method,[S6] the angles of guided modes (i.e., θ in Equation S1) that can excite the C-PR, the NGW-PR and the NFE-PR are within limited ranges, as shown in **Figures S2**a, **S2**b and **S2**c, respectively. The blue region represents the loss of the light energy and the excitation of the plasmonic resonance. A larger size of the blue region represents more guided modes that can excite the plasmonic resonance. Therefore, there are more olive intersections in the NGW-PR dispersion model compared to four magenta intersections in the C-PR dispersion model, as shown in **Figure S3**a. This indicates the FWHM of the NGW-PR is larger than that of the C-PR, as shown by the olive curve in **Figure S1**b. There are fewer blue intersections in the NFE-PR dispersion model, as shown in **Figure S3**b. This indicates the FWHM of the NFE-PR is smaller than that of the C-PR, as shown by the blue curve in **Figure S1**b.

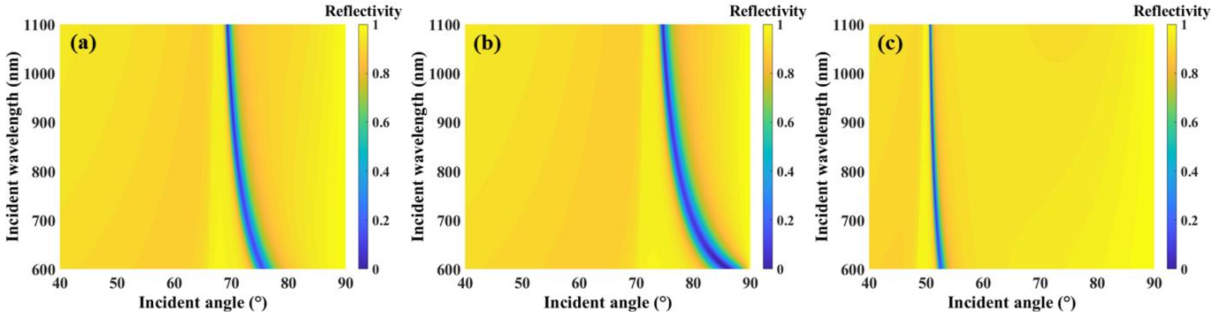

Figure S2. Angle ranges corresponding to the excitation of a) the C-PR, b) the NGW-PR and c) the NFE-PR. For the C-PR, the refractive indices of the silica fiber core and the sample layer are 1.4580 and 1.333, respectively. For the NGW-PR, the RI of the hybrid sample layer is approximately 1.373. For the NFE-PR, the RI of the hybrid fiber core is approximately 1.78. The metal layer is the Au layer.

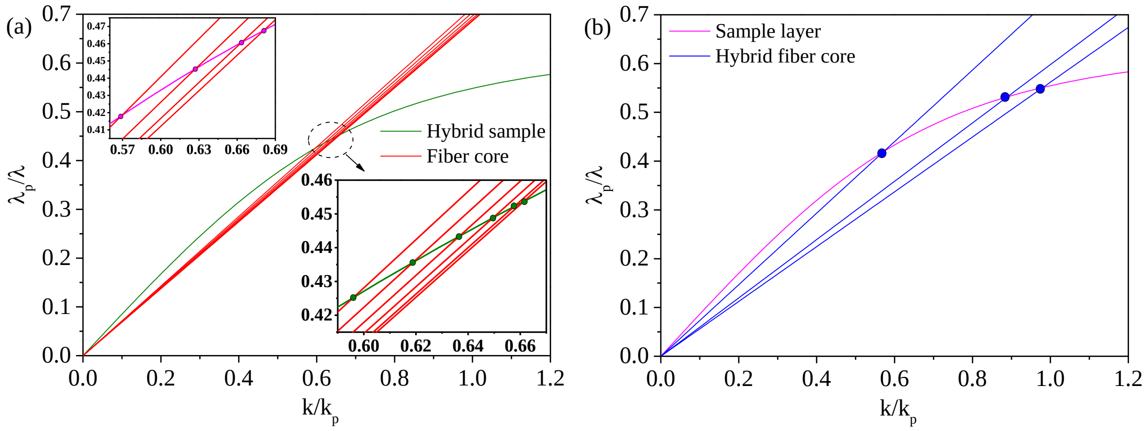

Figure S3. Dispersion models expressing the FWHM of a) the NGW-PR and b) the NFE-PR. Inset: the intersections in the C-PR dispersion model.

The second determinant of the FWHM is the radiation loss and the propagation distance of surface plasmon polaritons (SPPs).[S7] For the NGW-PR, SPPs propagate along the interface between the metal layer and the dielectric layer. The dielectric layer formed by a type of sheet-like two dimensional nanomaterial[S8] will significantly increase the radiation loss of SPPs. This also indicates a larger FWHM. For the NFE-PR, SPPs propagate along the interface between the metal layer and the sample layer with a smaller radiation loss. Meanwhile, the intense transfer of electrons between the dielectric layer and the metal layer enhances the electric field intensity of SPPs. This allows a longer propagation distance of SPPs. Therefore, the FWHM of the NFE-PR is obviously smaller than that of the C-PR, which allows a higher accuracy of the spectral signal demodulation.

S1.3. Sensitivity

The sensitivity of the wavelength-interrogation optical fiber plasmonic resonance with a fixed angle of incidence is evaluated by the redshift of the resonance wavelength with the per unit change in the RI of the sample layer.[S9] The sensitivity S_λ is revised into Equation S4 according to Ref. [S10].

$$S_\lambda = \frac{d\lambda}{dn_s} = \frac{\varepsilon_{mr}^2}{\frac{n_s^3}{2}\left|\frac{d\varepsilon_{mr}}{d\lambda}\right| + \left(\varepsilon_{mr} + n_s^2\right)\frac{\varepsilon_{mr} n_s}{n_o}\frac{dn_o}{d\lambda}} \tag{S4}$$

where ε_{mr} is the real part of the dielectric constant of the metal layer, n_s and n_o are refractive indices of the (hybrid) sample layer and the (hybrid) fiber core. Since the variation of n_o with λ is marginal, the term of $dn_o / d\lambda$ is close to 0. Therefore, the first contributing factor in the sensitivity is ε_{mr} . Since $\varepsilon_{mr} < 0$, the smaller the ε_{mr} is, the higher the sensitivity will be.

Another contributing factor in the sensitivity is the overlap integral of the electric field intensity of SPPs on the upper surface of the metal layer.[S1] The electronic transfer between the dielectric layer and the metal layer can create a significant enhancement of the electric field intensity of SPPs. The degree of the enhancement depends on the electromagnetic characteristics (e.g., the dielectric constant, the carrier concentration, the carrier mobility, the resistivity) of coating materials. Therefore, both the NGW-PR and the NFE-PR have higher sensitivity than that of the C-PR. In addition, the doping of dispersive metal nanomaterials into the dielectric layer can produce higher sensitivity due to the formed hot spots[S11] between the dispersive metal nanomaterials and the continuous metal layer. Nevertheless, the scattering of the ray arising from the dispersive nanomorphology will increase the radiation loss of SPPs and broaden the FWHM.[S12] This makes it more difficult to demodulate the spectral signal of the NGW-PR. Since SPPs directly sense the surrounding medium in the NFE-PR, the above situation shows negligible degradation on the NFE-PR based detection.

S2. Finite Element Analysis Models of the Optical Fiber Plasmonic Resonance

S2.1. Metal Layer

In order to investigate the influence of different metal layers on spectral characteristics of the optical fiber plasmonic resonance, FEA models involving the side-polished silica fiber that can produce a larger evanescent field leakage,[S13] six types of metal layers and the sample layer have been built, as shown in **Figure S4**a. The transition metals (Ag, Au, Cu, Cr, Pt and Pd, Ag, Au and Cu are members of the same family of elements, Ag, Au, Pt and Pd are rare precious metals) commonly used in the development of plasmonic resonance sensors are employed as metal layers. The Ag layer, the Au layer, the Cu layer and the Pt layer are commonly used in the development of RI sensors based on the optical fiber plasmonic resonance.[S14] The Cr layer is commonly used to enhance the adhesion of the metal layer to the surface of the fiber substrate.[S15] The Pd layer is commonly used in the development of the hydrogen sensor based on the optical fiber plasmonic resonance.[S16] Dielectric constants for six types of metal layers based on the Lorentz-Drude oscillator model[S17] are shown in **Figure S5**. Loss spectra of six types of fiber plasmonic resonances, which are derived from fundamental mode fields, can be used to analyze spectral characteristics,[S4] are shown in **Figure S6**.

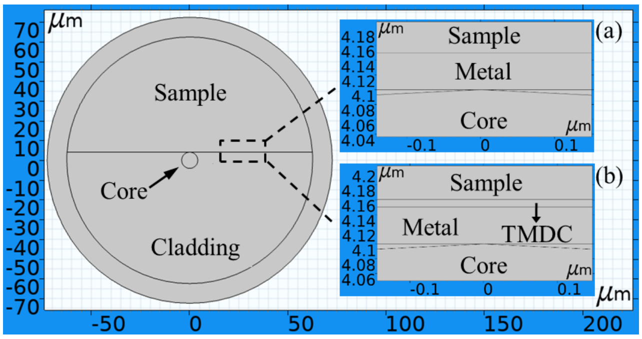

Figure S4. FEA models of (a) the fiber plasmonic resonance and (b) the TMDC-modified fiber plasmonic resonance. Thicknesses of the metal layer and the TMDC layer are 50 nm and 10 nm, respectively. The refractive index and the size of the optical fiber can be found in Ref. [S4].

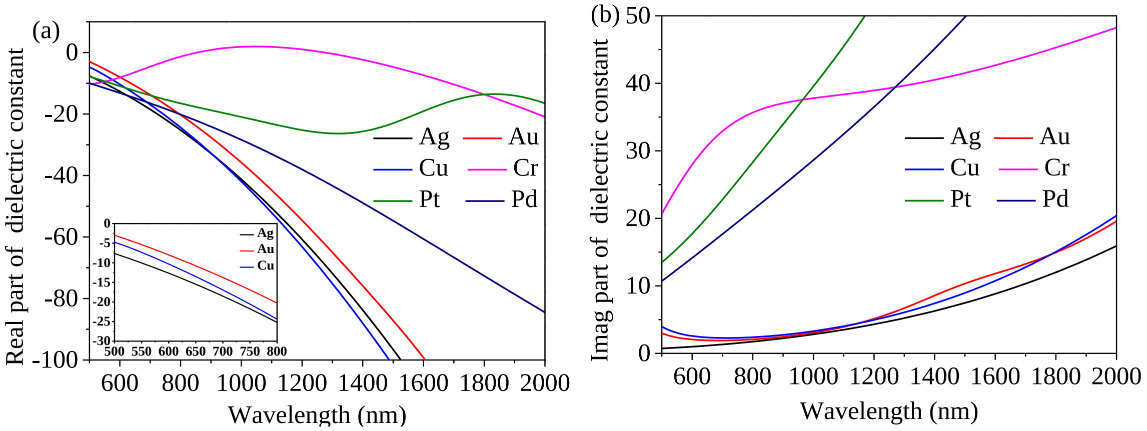

Figure S5. a) Real parts and b) imaginary parts of dielectric constants for six types of metal layers.

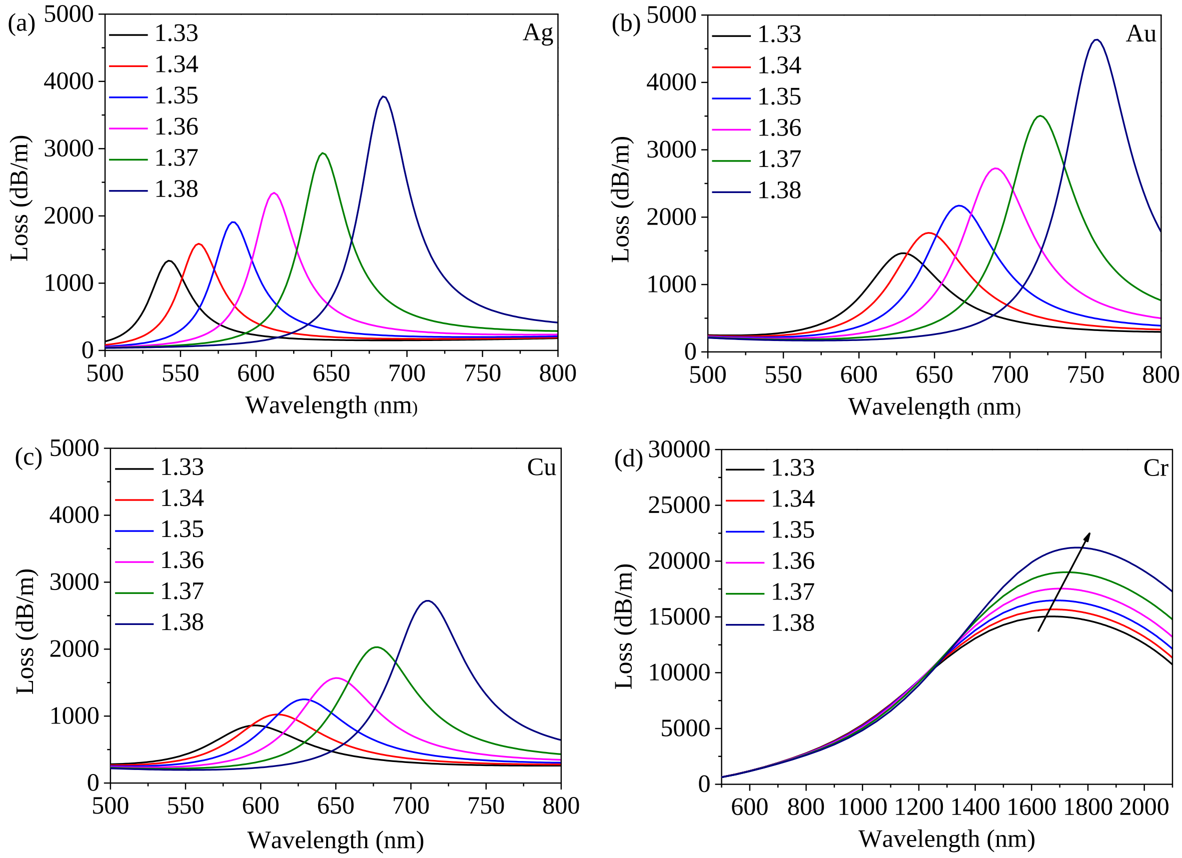

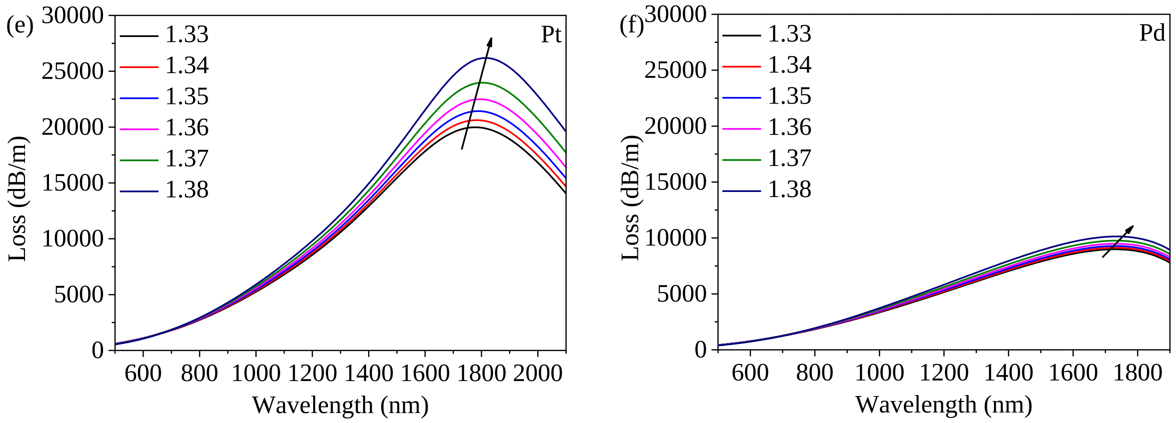

Figure S6. Loss spectra of six types of fiber plasmonic resonances corresponding to different refractive indices.

As shown in **Figure S7**a, for Ag, Cu, Au, Pd and Pt, the larger the real part of the dielectric constant is (wavelength range: 500 nm-800 nm for Ag, Cu and Au; 800 nm-2000 nm for Pd and Pt), the more obvious redshift the resonance wavelength exhibits; for Ag, Au, Pd and Pt, the larger the imaginary part of the dielectric constant is (wavelength range: 500 nm-800 nm for Ag and Au; 800 nm-2000 nm for Pd and Pt), the larger the resonance depth is. The sensitivity and the FWHM of such six types of fiber plasmonic resonances corresponding to each RI point are shown in **Figures S7**b and **S7**c, respectively. It is found that fiber plasmonic resonances constructed by noble metal layers possess higher sensitivity and smaller FWHM compared to those constructed by transition metal layers.

The FOM that can comprehensively evaluate the spectral characteristic of the fiber plasmonic resonance is given as follows:[S9]

$$FOM = \frac{S_{AV.}}{FWHM_{AV.}} \tag{S5}$$

where $S_{AV.}$ and $FWHM_{AV.}$ represent the average sensitivity and the average FWHM of the fiber plasmonic resonance, respectively. The DCR, that can evaluate the influence of differences between real parts and imaginary parts of dielectric constants for coating materials on spectral characteristic of the fiber plasmonic resonance, is defined as follows:

$$DCR = \frac{\left|\sum \varepsilon_r(\lambda)\right|}{\left|\sum \varepsilon_i(\lambda)\right|} \tag{S6}$$

where $\sum \varepsilon_r(\lambda)$ and $\sum \varepsilon_i(\lambda)$ represent the sum of real parts and the sum of imaginary parts for dielectric constants within a specific wavelength range, respectively. The DCR of such six types of metal layers (wavelength range: 500 nm - 800 nm for Ag, Au and Cu; 500 nm - 2000 nm for Cr, Pt and Pd) and the FOM of such six types of fiber plasmonic resonances are shown

in **Figure S7**d. It is found that the closer the DCR of the metal is to 1, the lower the FOM of the resonance excited by this type of metal layer is.

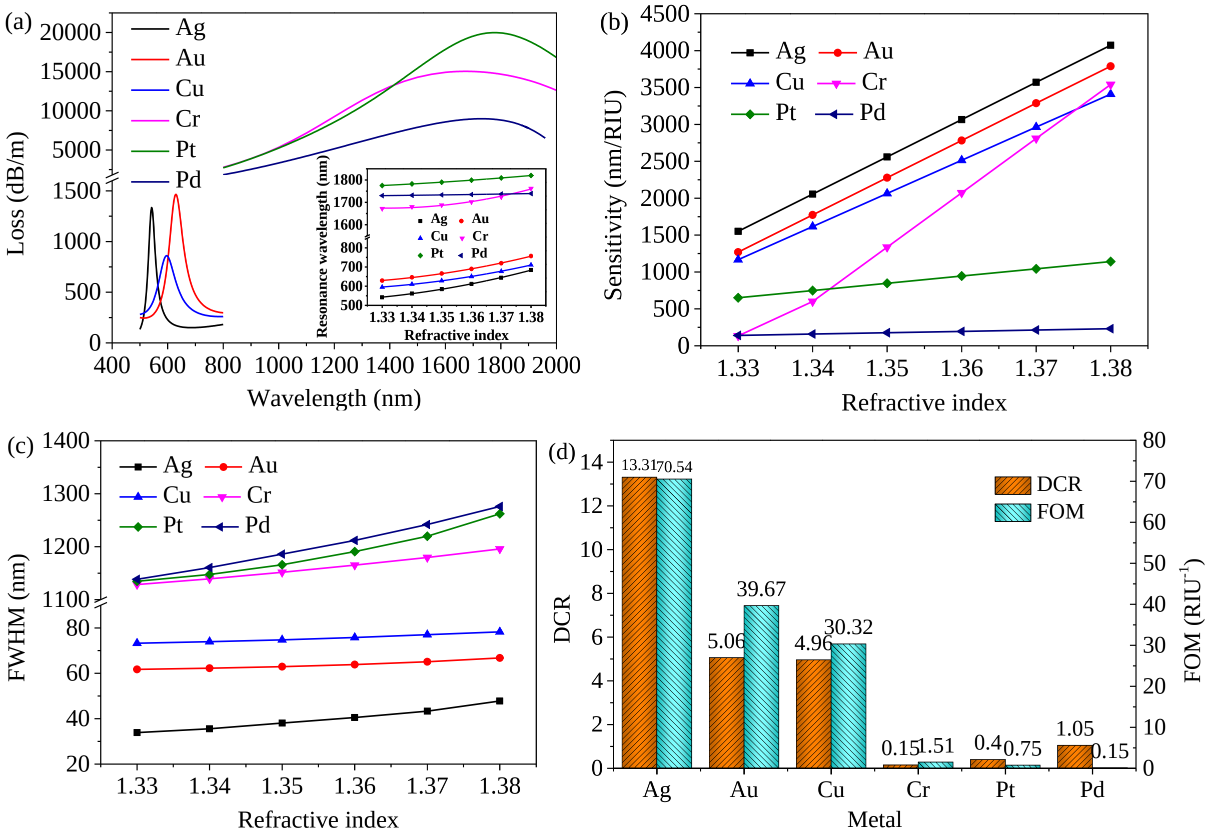

Figure S7. a) Loss spectra of six types of fiber plasmonic resonances corresponding to a sample RI of 1.33. Inset: binomial fitting curves of RI points and resonance wavelengths belonging to above six types of resonances. The tangent slope of each RI point in the curve is taken as the sensitivity of each RI point. b) The sensitivity and c) the FWHM of six types of resonances corresponding to different RI points. The average of the sensitivity and the FWHM of five RI points belonging to each resonance represent the average sensitivity and the average FWHM, respectively. d) The DCR of six types of metal layers and the FOM of corresponding six types of resonances.

S2.2. Dielectric Layer

In order to investigate the influence of different dielectric layers on spectral characteristics of the optical fiber plasmonic resonance, FEA models based on the Au layer, four types of dielectric layers (MoS_2, $MoSe_2$, WS_2, WSe_2, in **Figure S8**) and the sample layer have been built, as shown in **Figure S4**b. In the practical fabrication process, the thickness of the dielectric layer is usually on the order of ten nanometers, and the thickness of bulk TMDCs can achieve this level. Therefore, dielectric constants for four types of bulk TMDCs are calculated based on the first-principles calculations, the density functional theory and the pseudopotential plane wave method,[S18] as shown in **Figure S9**. Corresponding complex

refractive indices of four types of TMDC layers (in **Figure S10**) are interpolated into FEA models in order to achieve good agreements with experimental results. Loss spectra of four types of TMDC-modified fiber plasmonic resonances are shown in **Figure S11**.

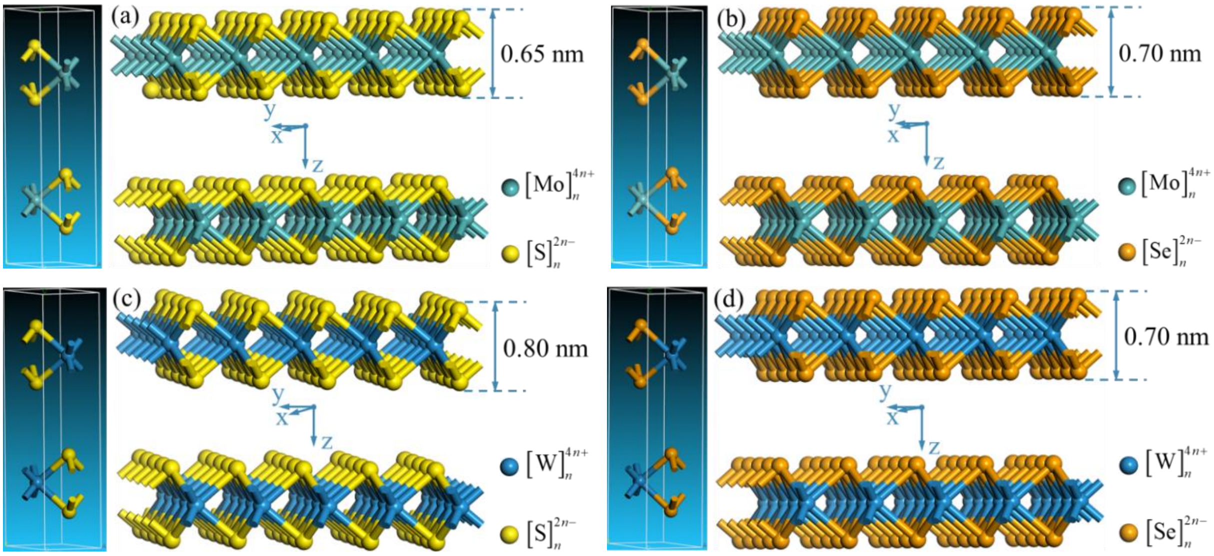

Figure S8. Schematic of unit cells and bi-layer structures for four types of TMDCs.

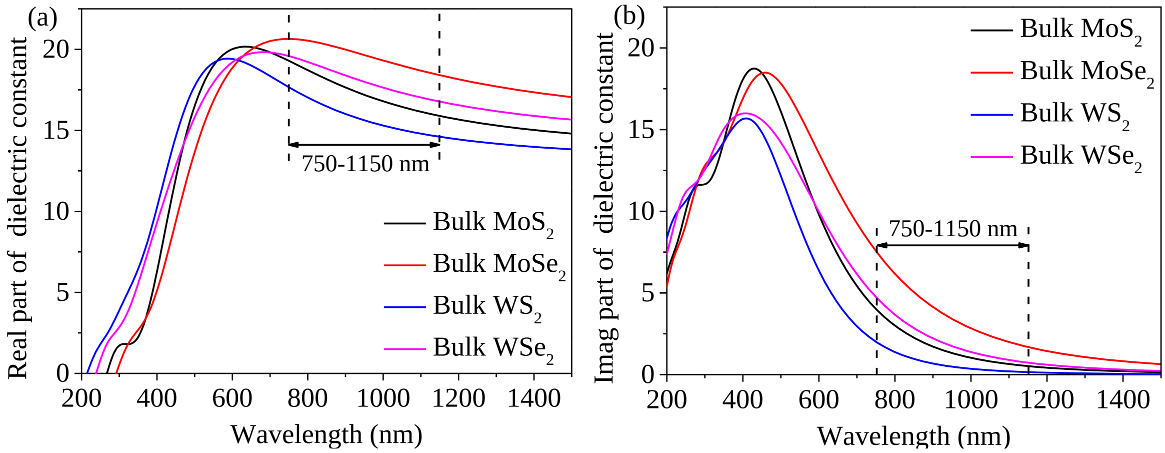

Figure S9. a) Real parts and b) imaginary parts of dielectric constants for four types of bulk TMDCs.

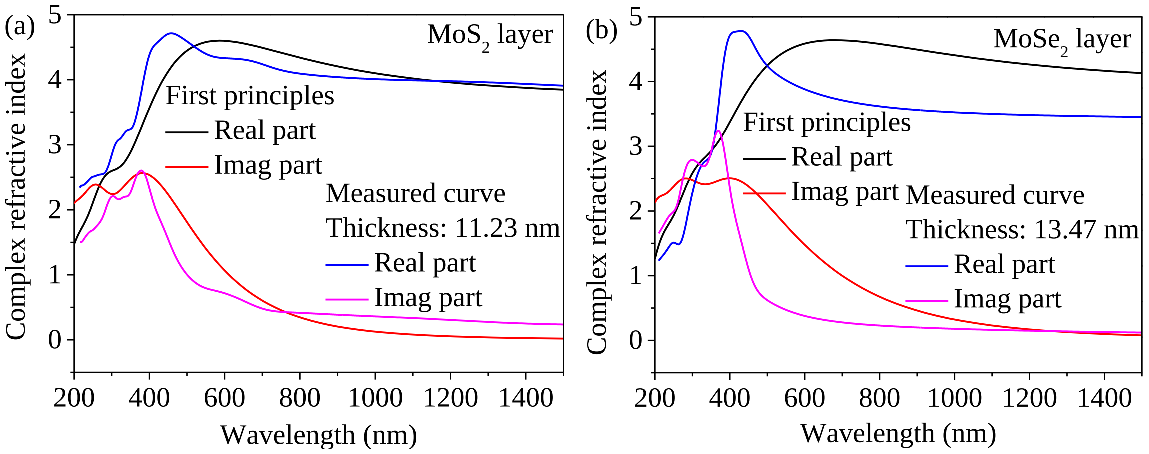

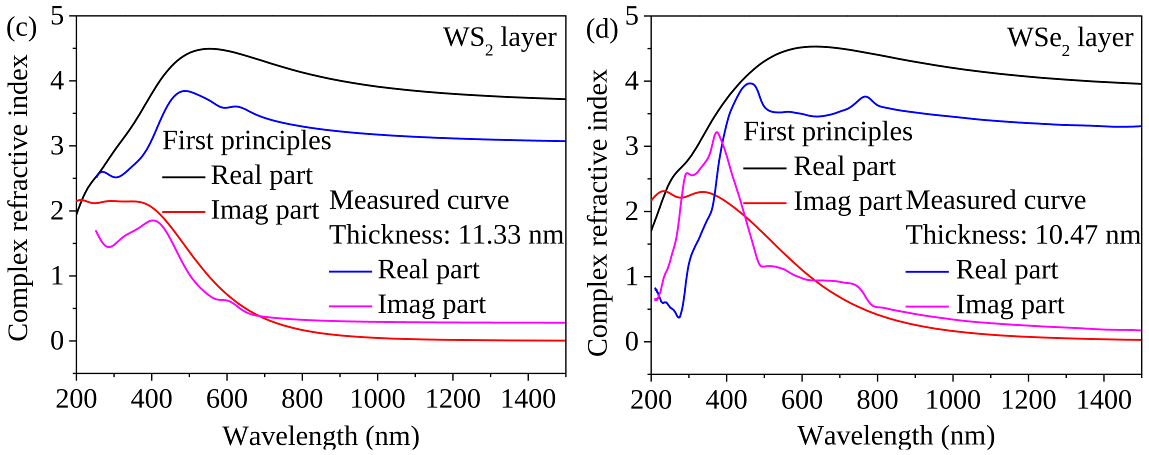

Figure S10. Complex refractive indices for four types of TMDC layers. The real part and the imaginary part represent the RI and the extinction coefficient, respectively. The measured curves are affected by various factors, e.g., film quality, fitting method, and thereby the measured curves are used for comparison only. Variations of measured curves obtained by an ellipsometer (M-2000V, J.A. Woollam Co., USA, 370 nm-1690 nm) are similar to those of calculated curves.

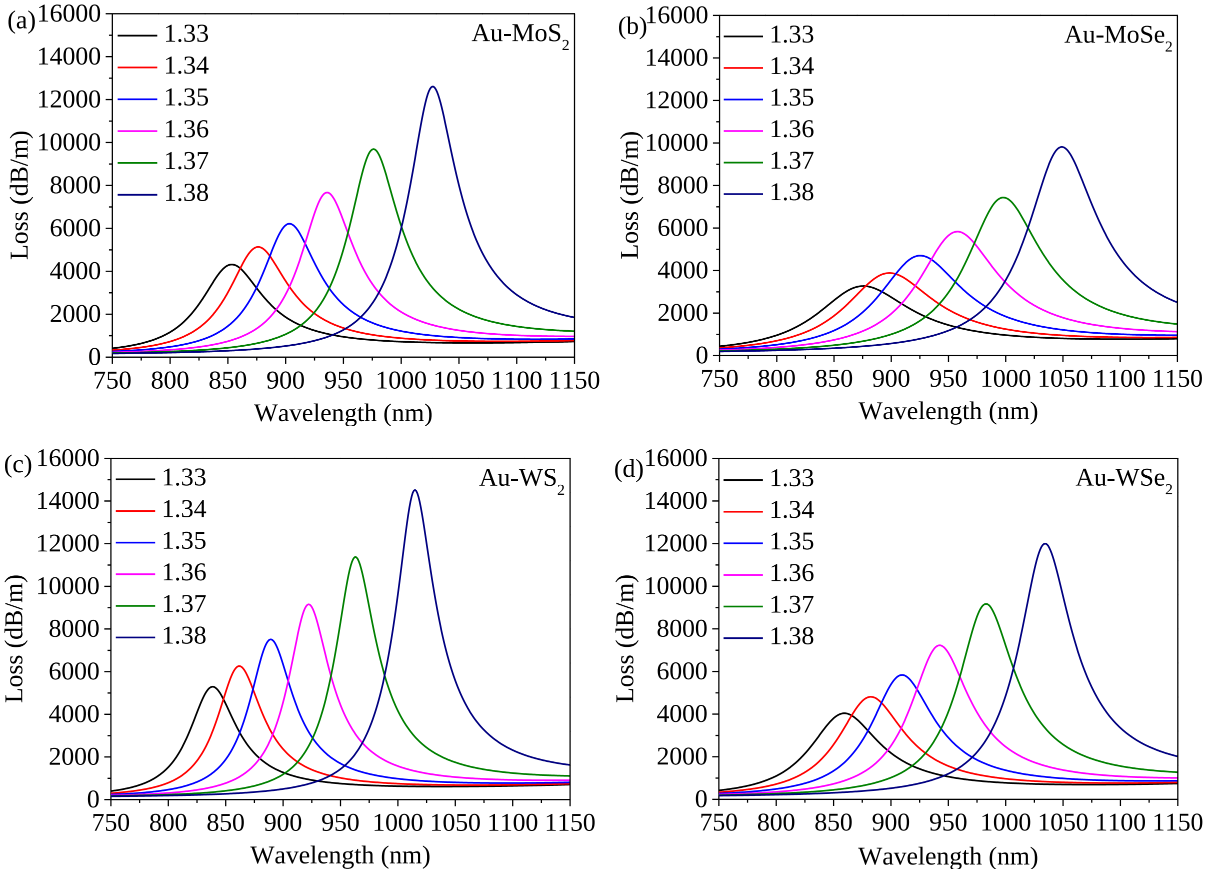

Figure S11. Loss spectra of four types of TMDC-modified fiber plasmonic resonances corresponding to different refractive indices.

Within the wavelength range of 750 nm-1000 nm, for such four types of TMDCs, the larger the real part of the dielectric constant is, the more obvious redshift the resonance

wavelength exhibits; the smaller the imaginary part of the dielectric constant is, the larger the resonance depth is, as shown in **Figure S12**a. The sensitivity and the FWHM of such four types of TMDC-modified fiber plasmonic resonances corresponding to each RI point are shown in **Figures S12**b and **S12**c. The DCR of such four types of TMDCs (wavelength range: 750 nm-1150 nm) and the FOM of such four types of resonances are shown in **Figure S12**d. It can be found that, the larger the DCR is, the higher the FOM of the resonance modified by this type of TMDC layer is. Moreover, the average sensitivity of fiber plasmonic resonances modified by WX_2 (X=S or Se) is higher than that of resonances modified by MoX_2 (X=S or Se), as shown by the inset in **Figure S12**a. This can be explained from electron configurations of Mo ([Kr] $4d^5 5s^1$) and W ([Xe] $4f^{14} 5d^4 6s^2$). According to the Hund's rule,[S19] the valence electron configuration of Mo is half-full, which has lower energy compared to that of W. Therefore, the chemical properties of WX_2 are more active.

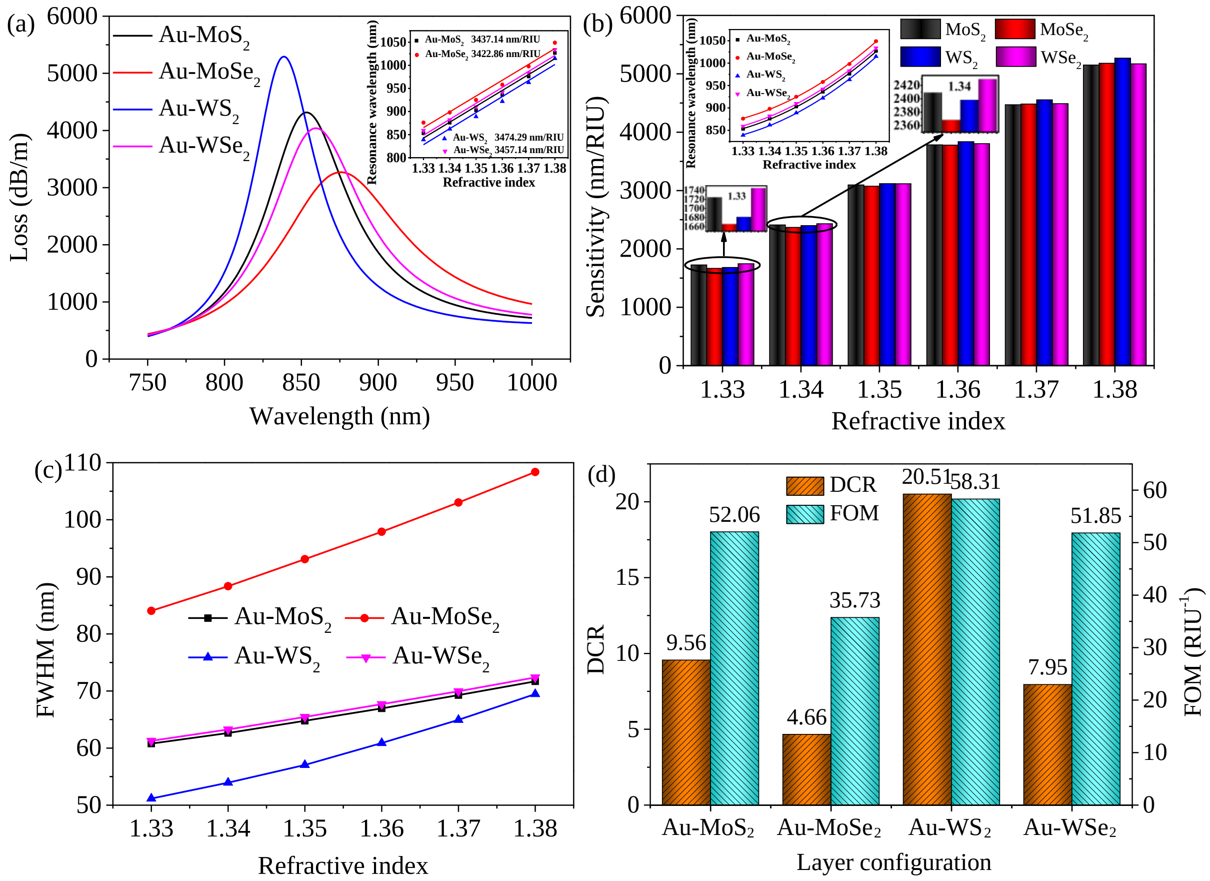

Figure S12. a) Loss spectra of four types of TMDC-modified fiber plasmonic resonances corresponding to a sample RI of 1.33. Inset: linear fitting of RI points and resonance wavelengths belonging to four types of resonances, and the slope of the fitted line represents the average sensitivity of each resonance. b) The sensitivity of four types of resonances corresponding to different RI points. Inset: binomial fitting curves of RI points and resonance wavelengths of four types of resonances, the tangent slope of each RI point in the curve is taken as the sensitivity of each RI point. c) Variations of the FWHM of four types of resonances with RI points. The average value of the FWHM corresponding to five RI points represents the average

FWHM of each resonance. d) The DCR of four types of TMDCs and the FOM of corresponding four types of resonances.

S3. The Optimized Configuration of the Antibody Coupling

The antibody coupling in optical fiber plasmonic resonance biosensors is mainly achieved by amino groups in antibody protein molecules. Currently popular antibody coupling agents include carboxyl-functionalized nanomaterials and the dopamine. The influence of types of antibody coupling agents (the carboxyl functional group and the dopamine) and dopamine coupling conditions (the alkalinity and the self-polymerization time) on the detection of the AFP has been investigated in this Section.

A fiber NGW-PR immunoprobe with a sensing structure of fiber core/Au layer/carboxylated WSe_2@AuNSs/CAbs has been developed for the detection of the AFP. The carboxyl functional groups on the surface of the WSe_2 layer are activated by the EDC and the NHS. The activated carboxyl groups can bind to amino groups in antibodies.[S20] The redshift of the resonance wavelength obtained by the immunoprobe is shown in the inset in **Figure S13**a and **Table S1**. Fiber NFE-PR immunoprobes with the same sensing structure of fiber core/WSe_2@AuNSs/Au layer/PDA layer/CAbs have also been developed for the detection of the AFP. The resonance wavelength redshifts of the immunoprobes obtained under different coupling conditions are shown in the inset in **Figure S13**b and **Table S1**.

It is found that a more obvious redshift of the resonance wavelength is obtained when the pH of Tris buffer is 8.5. The reason is that too strong or too weak alkalinity is not conducive to the generation of quinone groups and reduces the efficiency of the antibody-immobilization. It can also be found that a longer self-polymerization time is more beneficial to the detection of the AFP. This is because a long self-polymerization time allows more dopamine to polymerize to the surface of the Au layer. The formed PDA layer can be regarded as a dielectric layer with a high RI, as shown in **Figure S14**. This enables the fiber NFE-PR immunoprobe to have higher sensitivity and better biocompatibility. Meanwhile, there is no obvious difference in redshifts obtained with self-polymerization times of 120 mins and 180 mins. This indicates that the redshift will not increase infinitely with the increment of the self-polymerization time due to the limited number of antigens. In addition, a too long self-polymerization time will lead to a granular agglomeration of the dopamine, and will severely broaden the FWHM to reduce the accuracy of the resonance signal demodulation. To sum up, the appropriate pH of the Tris buffer and the self-polymerization time are 8.5 and 120 mins, respectively.

The initial resonance wavelength (the blue curve in **Figure S13**a) of the fiber NGW-PR immunoprobe is similar to that (the blue curve in **Figure S13**b) of the fiber NFE-PR immunoprobe with optimized coupling conditions in the detection of the AFP. This ensures that two types of immunoprobes have similar sensitivity. Therefore, the more obvious redshift of the resonance wavelength obtained by the fiber NFE-PR immunoprobe is mainly due to the better biocompatibility of the dopamine compared to carboxylated WSe_2 nanosheets. The fiber NFE-PR immunoprobe with the antibody coupling agent of the dopamine is more suitable for the detection of the AFP. This achieves a good agreement with analyses in Section S1.

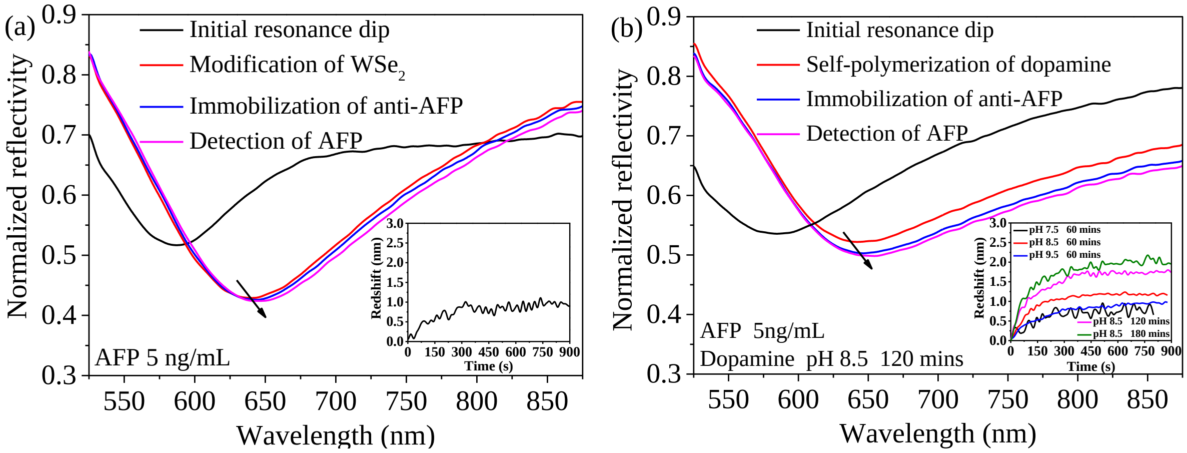

Figure S13. a) Resonance spectra of the fiber NGW-PR immunoprobe before the modification of the carboxylated WSe_2@AuNSs layer (black curve), after the modification of the carboxylated WSe_2@AuNSs layer (red curve), after the immobilization of CAbs (blue curve) and after the detection of the AFP (magenta curve). Inset: the resonance wavelength redshift obtained in detecting AFP with the immunoprobe. b) Resonance spectra of the fiber NFE-PR immunoprobe before the self-polymerization of the dopamine (black curve), after the self-polymerization of the dopamine (red curve), after the immobilization of CAbs (blue curve) and after the detection of the AFP (magenta curve). Inset: resonance wavelength redshifts obtained in detecting AFP with the immunoprobes under different dopamine coupling conditions.

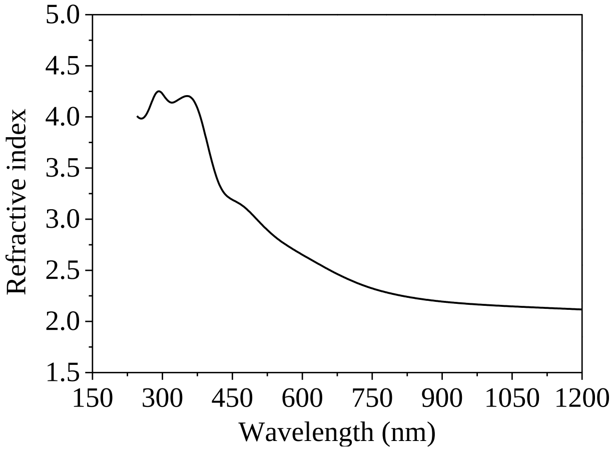

Figure S14. Refractive indices of the PDA layer and the silica fiber core.

Table S1. The influence of different antibody-coupling conditions on the detection of the AFP.

Type of the immunoprobe	Type of the coupling agent	pH of the Tris buffer solution	Self-polymerization time of the dopamine (min)	Concentration of the AFP (ng/mL)	Redshift of the resonance wavelength (nm)
NGW-PR	Carboxyl groups on the WSe_2 layer	_	_	5	**1.02**
NFE-PR	Dopamine	7.5	60		0.81
		8.5	60		1.17
		9.5	60		0.96
		8.5	120		**1.79**
		8.5	180		1.87

S4. The Discussion on the Non-specific Deposition of the Antigen

S4.1 The Discussion on the Intra-batch Difference of Immunoprobes

The PBS-prepared alpha-fetoprotein (AFP) samples are detected for the calibration of the immunoprobe, and the human serum samples are measured for the demonstration of the clinical applicability of the immunoprobe. The concentrations of PBS-prepared AFP samples include 0 ng/mL (blank sample), 0.1 ng/mL, 0.2 ng/mL, 0.5 ng/mL, 1 ng/mL, 2 ng/mL, 5 ng/mL, 10 ng/mL, 20 ng/mL, 40 ng/mL, 60 ng/mL, 80 ng/mL and 100 ng/mL. These samples are detected by thirteen immunoprobes, fabricated in the same batch, with similar performance. This avoids the degradation of the sensing performance of the immunoprobe due to the dissociation and the wash of the antigen-antibody complex when the samples with different concentrations are measured using the same sensing unit.

The location of the resonance dip of the optical fiber plasmonic resonance is closely related to the sensitivity and the detection limit of the developed sensor. The resonance dips and the resonance wavelengths of immunoprobes fabricated in the same batch are shown in **Figures S15**a **and S15**b, respectively. The variation of the depth of the resonance dip arises from the fluctuation of the intensity of the light source. The locations of resonance dips are highly coincident, and the standard deviation of resonance wavelengths demodulated at the same time node is 0.1946 nm. This indicates a moderate intra-batch difference of immunoprobes and the good precision of resonance wavelength redshifts for the detection of AFP samples.

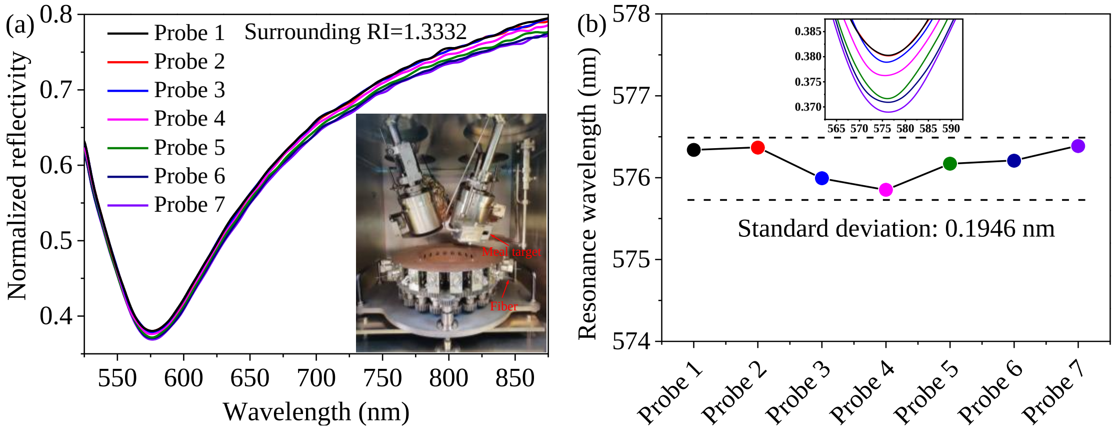

Figure S15. a) Resonance dips collected by 7 probes at the 30th second after the immersion in the deionized water. Inset: the optical fiber magnetron sputtering vacuum chamber, up to 20 probes can be fabricated simultaneously in the same batch. b) The resonance wavelengths of 7 probes. Inset: the detailed view of the bottom of resonance dips.

S4.2 The Introduction of the PBS Immersion After the Detection of the AFP

In this experiment, the step of the PBS immersion is introduced after the detection of the AFP sample of 80 ng/mL. This guarantees that the refractive index is measured before and after the binding event in the same condition, i.e., in the PBS solution. The experimental result is shown in the **Figure S16**.

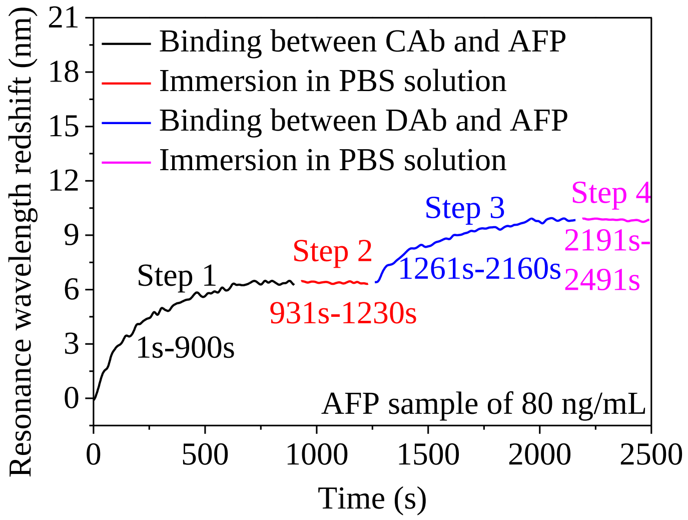

Figure S16. The redshift of the resonance wavelength for the detection of the AFP sample of 80 ng/mL with the step of the PBS immersion. The final redshift is 9.8030 nm calculated from the mean value of redshifts over the last 50 seconds.

Step 1 represents the resonance wavelength redshift for the dynamic binding between the primary antibody (i.e., the capture antibody (CAb)) and the AFP.

Step 2 denotes that the immunoprobe is immersed in the PBS solution.

Step 3 refers to the redshift for the signal enhancement via the secondary antibody (i.e., the detection antibody (DAb)).

Step 4 indicates that the immunoprobe is immersed in the PBS solution again.

The cumulative resonance wavelength redshift is 9.8030 nm. Our work is focused on the dynamic binding between the antibody and the antigen. Therefore, only Steps 1 and 3 are included in **Figure** 5a in our manuscript, and the obtained resonance wavelength redshift is 9.8499 nm (orange curves **Figure** 5a corresponding to 80 ng/mL). The difference between the two redshifts (0.0469 nm) is within the range of the error caused by demodulation algorithms and the intra-batch difference of immunoprobes. This indicates that the PBS immersion has a marginal influence on the calibration of the immunoprobe and the calculation of the limit of detection (LOD). This is because the number of antibodies is excessive compared to the number of antigens in the sample with a concentration at the level of ng/mL. This allows the antigens to fully bind to the antibodies. Therefore, the non-specific deposition/binding of the antigen has a marginal impact on the redshift signal arising from the specific interaction.

The introduction of Step 2 and Step 4 will produce additional procedures and operation time in the detection. This is not beneficial to meeting requirements of the rapid detection and the easy operation in the early screening of tumors. Therefore, the step of the PBS immersion has not been applied in the detection of the AFP in our work.

The specificity of the immunoprobe has been discussed in Section 2.2.2. It can be found that the resonance wavelength redshift arising from the control sample is obviously smaller than that arising from the target sample, as seen in **Figure 7**b. This also indicates the marginal effect of the non-specific binding on the detection of the serum sample and also demonstrates the applicability of the calibration curve in the detection of the serum sample.

S5. Linear Regression Analysis

Linear regression is a method for analyzing the linear correlation between the independent variable (x_i) and the dependent variable (x_i) in applied mathematical statistics.[S21] The univariate linear regression model and the prediction interval are given by Equations S7 and S8, respectively. The relevant parameters used in the calculation are shown in **Tables S2** and **S3**, respectively.

$$y = \beta_0 + \beta_1 x \quad \text{(S7)}$$

$$\begin{cases} \left[y - \delta(x), y + \delta(x) \right] \\ \delta(x) = \sigma t_{1-\frac{\alpha}{2}}(n-2)\sqrt{1 + \frac{1}{n} + \frac{\left(x_0 - \overline{x_i}\right)^2}{S_{xx}}} \end{cases} \quad \text{(S8)}$$

Table S2. Concentrations of the AFP in serum samples based on calibration curves of the fiber NFE-PR immunoprobe and the magnetic nanoparticle chemiluminescence kit (ng/mL).

AFP-spiked serum sample	Fiber plasmonic immunoprobe (x_i)		MNCK (y_i)
	Logistic	Langmuir	
Sample 1 ($i=1$)	1.93	2.03	2.13
Sample 2 ($i=2$)	5.02	4.96	5.74
Sample 3 ($i=3$)	11.59	11.57	10.36
Sample 4 ($i=4$)	21.37	21.62	19.87
Sample 5 ($i=5$)	37.56	37.62	41.28

Table S3. Key parameters for calculating the linear regression equations and the prediction intervals.

Parameter	Logistic	Langmuir	Note
$\overline{x_i} = \frac{1}{n}\sum_{i=1}^{n} x_i$	15.4940	15.5600	Average of sample data
$\overline{y_i} = \frac{1}{n}\sum_{i=1}^{n} y_i$	15.8760	15.8760	Average of sample data

$S_{xx}=\sum_{i=1}^{n}x_i^2-n\overline{x_i}^2$	830.3637	834.7082	Self-definited variable
$S_{xy}=\sum_{i=1}^{n}x_iy_i-n\overline{x_i}\,\overline{y_i}$	898.1831	900.0497	Self-definited variable
$S_{yy}=\sum_{i=1}^{n}y_i^2-n\overline{y_i}^2$	983.4325	983.4325	Self-definited variable
$\beta_1=\frac{S_{xy}}{S_{xx}}$	1.0817	1.0783	Least squares estimation
$\beta_0=\overline{y_i}-\beta_1\overline{x_i}$	-0.8834	-0.9020	Least squares estimation
$\sigma=\frac{S_{yy}-\beta_1S_{xy}}{n-2}$	1.9912	2.0758	Variance
$R^2=\beta_1^{\ 2}\frac{S_{xx}}{S_{yy}}$	98.7909%	98.6856%	Correlation coefficient
Number of samples $n=5$, t-distributed quantile $t_{1-\alpha/2}\left(n-2\right)$ =3.1824, confidence coefficient $1-\alpha=95\%$			

Supplementary Figures

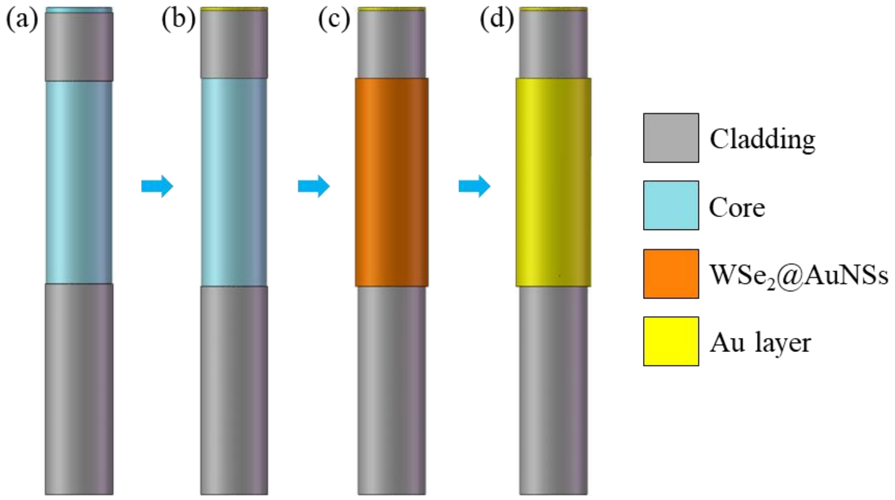

Figure S17. Schematic of a) the preprocessing of the optical fiber, b) the sputtering of the Au reflective mirror, c) the coating of the WSe_2@AuNSs layer and d) the sputtering of the Au layer.

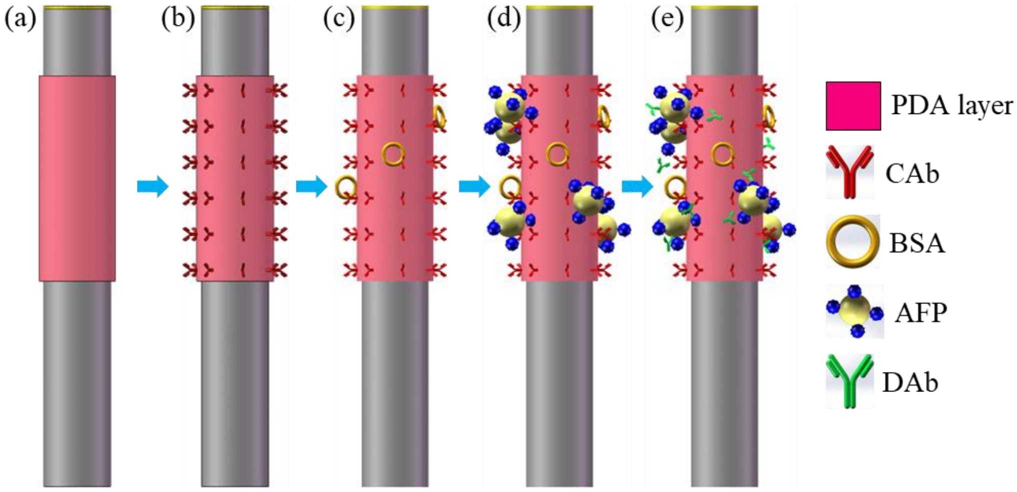

Figure S18. Schematic of a) the self-polymerization of the dopamine, b) the immobilization of CAbs, c) the BSA blocking in the surface passivation, d) the immunoassay of the AFP and e) the double-antibody sandwich immunoassay of the AFP.

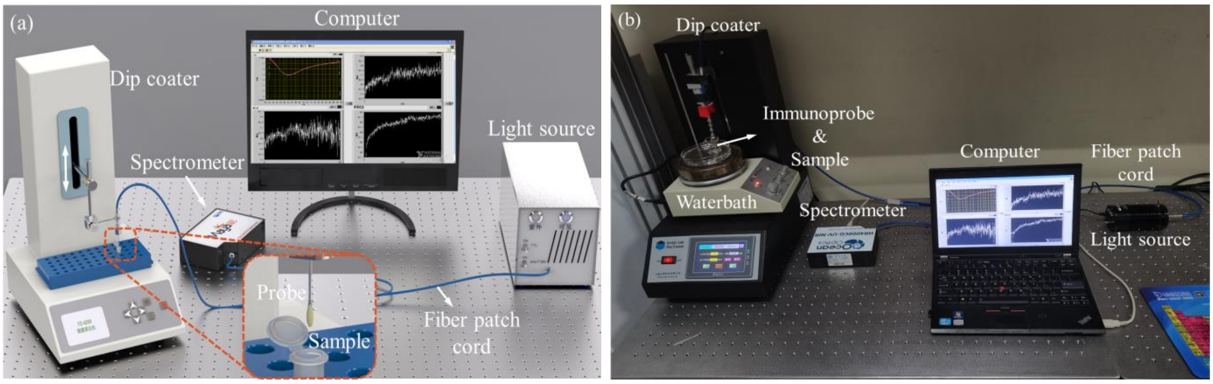

Figure S19. a) Schematic and b) realistic image of the experimental setup.

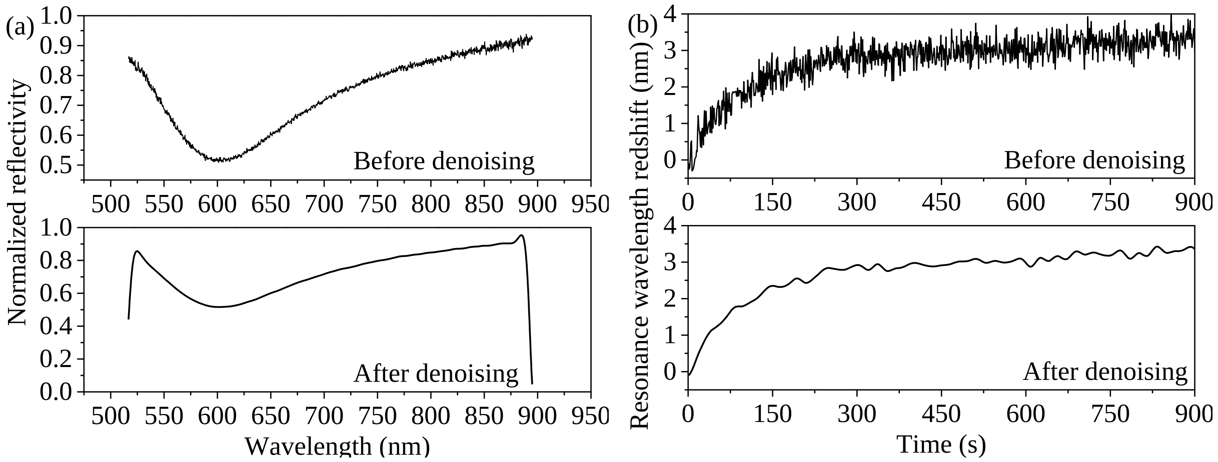

Figure S20. The real-time demodulation and the noise suppression of spectral signals: a) the resonance dip, b) the redshift of the resonance wavelength.

References

[1] A. S. halabney, I. Abdulhalim, Sensitivity-enhancement methods for surface plasmon sensors. *Laser Photonics Rev.* **2011**, 5, 571.

[2] J. J. Lao, P. Sun, F. Liu, X. J. Zhang, C. X. Zhao, W. J. Mai, T. Guo, G. Z. Xiao, J. Albert, In situ plasmonic optical fiber detection of the state of charge of supercapacitors for renewable energy storage. *Light-Sci. Appl.* **2018**, 7, 34.

[3] S. Shi, L. B. Wang, R. X. Su, B. S. Liu, R. L. Huang, W. Qi, Z. M. He, A polydopamine-modified optical fiber SPR biosensor using electroless-plated gold films for immunoassays. *Biosens. Bioelectron*. **2015**, 74, 454.

[4] J. J. Jing, K. Liu, J. F. Jiang, T. H. Xu, S. Wang, T. G. Liu, Highly sensitive and stable probe refractometer based on configurable plasmonic resonance with nano-modified fiber core. *Opto-Electron. Adv.* **2023**, 6, 220072.

[5] M. Kanso, S. Cuenot, G. Louarn, Sensitivity of optical fiber sensor based on surface plasmon resonance: modeling and experiments. *Plasmonics*. **2008**, 3, 49.

[6] W. M. Zhao, Q. Wang, Analytical solutions to fundamental questions for lossy mode resonance. *Laser Photonics Rev*. DOI: 10.1002/lpor.202200554.

[7] B. Dastmalchi, P. Tassin, T. Koschny, C. M. Soukoulis, A new perspective on plasmonics: confinement and propagation length of surface plasmons for different materials and geometries. *Adv. Opt. Mater*. **2016**, 4, 177.

[8] Z. Y. Hao, Y. Liu, Z. H. Zhao, Q. Wang, Highly sensitive gold-film surface plasmon resonance (SPR) sensor employing germanium selenide (GeSe) nanosheets. *Instrum. Sci. Technol.* **2022**, 50, 577.

[9] A. B. Socorro-Leránoz, D. Santano, I. Del. Villar, I. R. Matias, Trends in the design of wavelength-based optical fibre biosensors (2008-2018). *Biosensors and Bioelectronics: X*. **2019**, 1, 100015.

[10] J. Homola, I. Koudela, S. S. Yee, Surface plasmon resonance sensors based on diffraction gratings and prism couplers: sensitivity comparison. *Sensor. Actuat. B-Chem.* **1999**, 54, 16.

[11] H. Harutyunyan, A. B. F. Martinson, D. Rosenmann, L. K. Khorashad, L. V. Besteiro, A. O. Govorov, G. P. Wiederrecht, Anomalous ultrafast dynamics of hot plasmonic electrons in nanostructures with hot spots. *Nat. Nanotechnol.* **2015**, 10, 770.

[12] Z. H. Zhao, Q. Wang, Gold nanoparticles (AuNPs) and graphene oxide heterostructures with gold film coupling for an enhanced sensitivity surface plasmon resonance (SPR) fiber sensor. *Instrum. Sci. Technol*. **2022**, 50, 530.

[13] Y. J. Wang, S. G. Li, M. Y. Wang, P. T. Yu, Refractive index sensing and filtering characteristics of side-polished and gold-coated photonic crystal fiber with a offset core. *Opt. Laser Technol*. **2021**, 136, 106759.

[14] N. K. Sharma, S. Shukla, V. Sajal, Surface plasmon resonance based fiber optic sensor using an additional layer of platinum: A theoretical study. *Optik* **2017**, 133, 43.

[15] B. J. Jin, D. N. Wang, Multimode fiber surface plasmon resonance sensor based on a down-up taper. *Opt. Lett.* **2022**, 47, 5329.

[16] X. J. Zhang, S. S. Cai, F. Liu, H. Chen, P. G. Yan, Y. Yuan, T. Guo, J. Albert, In situ determination of the complex permittivity of ultrathin H-2-infused palladium coatings for plasmonic fiber optic sensors in the near infrared. *J. Mater. Chem. C.* **2018**, 6, 5161.

[17] A. D. Rakic, A. B. Djurisic, J. M. Elazar, M. L. Majewski, Optical properties of metallic films for vertical-cavity optoelectronic devices. *Appl. Optics*. **1998**, 37, 5271.

[18] M. R. Osanloo, M. L. Van de Put, A. Saadat, W. G. Vandenberghe, Identification of two-dimensional layered dielectrics from first principles. *Nat. Commun*. **2021**, 12, 5051.

[19] A. A. Khajetoorians, M. Valentyuk, M. Steinbrecher, T. Schlenk, A. Shick, J. Kolorenc, A. I. Lichtenstein, T. O. Wehling, R. Wiesendanger, J. Wiebe, Tuning emergent magnetism in a Hund's impurity. *Nat. Nanotechnol*. **2015**, 10, 958.

[20] T. M. Pan, C. W. Wang, W. C. Weng, C. C. Lai, Y. Y. Lu, C. Y. Wang, I. C. Hsieh, M. S. Wen, Rapid and label-free detection of the troponin in human serum by a TiN-based extended-gate field-effect transistor biosensor. *Biosens. Bioelectron*. **2022**, 201, 113977.

[21] J. Hong, A. Kawashima, N. Hamada, A simple fabrication of plasmonic surface-enhanced Raman scattering (SERS) substrate for pesticide analysis via the immobilization of gold nanoparticles on UF membrane. *Appl. Surf. Sci*. **2017**, 407, 440.
